# Exploring the self-efficacy and self-care-based stroke care model for risk factor modification in mild-to-moderate stroke patients

**DOI:** 10.3389/fneur.2023.1177083

**Published:** 2023-05-11

**Authors:** Al Rasyid, Uke Pemila, Siti Aisah, Salim Harris, Elvan Wiyarta, Marc Fisher

**Affiliations:** ^1^Department of Neurology, Faculty of Medicine, Universitas Indonesia, Dr. Cipto Mangunkusumo National Hospital, Jakarta, Indonesia; ^2^Directorate of Health Service Governance, Indonesian Ministry of Health, Jakarta, Indonesia; ^3^Department of Medical Surgery, Faculty of Nursing, Universitas Indonesia, Dr. Cipto Mangunkusumo National Hospital, Jakarta, Indonesia; ^4^Department of Medical Science, Faculty of Medicine, Universitas Indonesia, Dr. Cipto Mangunkusumo National Hospital, Jakarta, Indonesia; ^5^Beth Israel Deaconess Medical Center, Boston, MA, United States

**Keywords:** ischemic stroke, patient education, prevention, discharge education, Indonesia, self-confidence

## Abstract

**Context:**

The worldwide burden of stroke is projected to grow unless proper stroke education is implemented. Information alone cannot promote patient self-efficacy and self-care and reduce risk factors.

**Aim:**

This trial aimed to test self-efficacy and self-care-based stroke education (SSE) on changes in self-efficacy, self-care, and risk factor modification.

**Design, setting, and participants:**

This study is a single-center, double-blinded, interventional, two-arm randomized controlled trial with a 1- and 3-month follow-up in Indonesia. Between January 2022 and October 2022, 120 patients were prospectively enrolled from Cipto Mangunkusumo National Hospital, Indonesia. Participants were assigned using a computer-generated random number list.

**Intervention:**

SSE was given before discharge from the hospital.

**Primary outcome measure:**

Self-care, self-efficacy, and stroke risk score was measured 1 month and 3 months after discharge.

**Secondary outcome measure:**

Modified Rankin Scale, Barthel Index, and blood viscosity was measured at 1 month and 3 months after discharge.

**Results:**

A total of 120 patients (intervention *n* = 60; standard care *n* = 60) were randomized. In the 1st month, the intervention group showed a more significant change in self-care (4.56 [95% CI: 0.57, 8.56]), self-efficacy (4.95 [95% CI: 0.84, 9.06]), and stroke risk (−2.33 [95% CI:−3.19, −1.47]) compared to the controlled group. In the 3rd month, the intervention group also showed a more significant change in self-care (19.28 [95% CI: 16.01, 22.56]), self-efficacy (19.95 [95% CI: 16.61, 23.28]), and stroke risk (−3.83 [95% CI: −4.65, −3.01]) compared to the controlled group.

**Conclusion:**

SSE may boost self-care and self-efficacy, adjust risk factors, enhance functional outcomes, and decrease blood viscosity.

**Clinical trial registration:**

ISRCTN11495822.

## Introduction

Globally, stroke is the second major cause of mortality and the third most significant cause of disability-adjusted life years (DALY) loss (1). From 1990 to 2019, the incidence of stroke increased dramatically, with lower-income and lower-middle-income countries bearing the most significant global burden, accounting for 86% of fatalities and 89% of DALYs lost (1). This increase is also strongly associated with uncontrolled stroke risk factors, particularly modifiable risk factors such as blood pressure, blood sugar, cholesterol, body mass index (BMI), alcohol consumption, smoking, physical activity, and diet (2).

Despite advances in stroke treatment, stroke incidence and death have risen (3). The Global Burden of Diseases, Injuries, and Risk Factors Study on Stroke demonstrates that the global burden would increase without adequate patient stroke education (3). As a cost-effective preventive approach, global institutions are creating models for stroke education (4). Educational initiatives, mnemonics, stroke codes, and fast tracts continue to provide unsatisfactory results in reducing the burden of stroke (4). Stroke patients and their families cannot take sufficient “action” with insufficient “information” (5). This knowing–doing gap may result from patients' inability to apply “knowledge” (5). Uncertainty and discontinuity indicate poor self-efficacy and insufficient self-care in stroke patients (6). These values need to be addressed to bridge the knowing–doing gap (7).

The concept of self-care was developed by Orem (1991) and defined as “the practice of activities that maturing and mature persons initiate and perform, within time frames, on their behalf in the interests of maintaining life, healthful functioning, continuing personal development, and wellbeing” (8). Meanwhile, self-efficacy was introduced by Bandura (1977) which “refers to beliefs in one's capabilities to organize and execute the courses of action required to produce given attainments” (9). Due to the lengthy and continuous stroke recovery process, these two values may be crucial in treating stroke patients.

Several trials related to stroke education have been conducted, such as the DESERVE trial (10) and the multidisciplinary Stroke Education Program (11). However, these trials did not emphasize self-efficacy and self-care-based education. Eames et al. (12) have carried out self-efficacy-based education but less emphasized the role of self-care after stroke. Amiri et al. (13) also conducted a trial on self-efficacy and its relationship with self-management but did not provide a relationship with stroke risk factor modification. The latest trial, SPRINT INDIA, researched stroke prevention using a structured semi-interactive stroke prevention package, but this trial did not emphasize evaluating self-care and self-efficacy (14). Although several trials have examined similar interventions, and some have reported less significant results (15), the new intervention design must be implemented in various countries with diverse cultural, economic, and linguistic burdens. This is the novelty value of this research.

The Self-Care and self-efficacy On Risk Education for Stroke (SCORES) trial was done using the intervention of self-efficacy and self-care-based stroke education (SSE) to narrow the knowing–doing gap in stroke patients and enhance earlier studies. In line with the world's burden of stroke (3), the SCORES trial was conducted in Indonesia, a lower-middle-income country with a large population. In this study, we examined the hypothesis that SSE would boost patient self-efficacy and self-care, reduce modifiable risk variables, and enhance functional outcomes compared to standard care.

## Methods

### Trial design, setting, and participants

This study is a single-center, double-blinded, interventional, two-arm randomized controlled trial with a 1- and 3-month follow-up in Indonesia. Between January 2022 and October 2022, 120 patients were prospectively enrolled from Cipto Mangunkusumo National Hospital. Before random allocation, participants provided written informed consent and met inclusion and exclusion criteria. Using simple randomization, participants were allocated to either the intervention or standard care groups. A statistician without clinical background assigned participants using a computer-generated random number list. The allocation sequence was concealed behind sealed envelopes in the researcher's ward. The researcher allocated patients and recorded their information. The allocation group of the patient was not specified. To reduce bias, nurses who provided instruction were distinguished from those who provided routine ward care. The nurses who provided the education did not encounter the patients before initiating the discharge education programs. All study arms, regardless of randomization group, received a standard of care (monitor vital signs, daily consumption, daily medicines, and an adequate health care facility), self-care, self-efficacy, and stroke risk assessments (including blood pressure, anthropometric, and risk factor evaluations). At baseline, demographics, self-care, self-efficacy, stroke risk score, National Institutes of Health Stroke Scale (NIHSS), Barthel index, modified Rankin Scale (mRS), blood viscosity, hospital stay, and comorbidities were collected.

Patients with mild-to-moderate ischemic stroke based on a clinical definition of focal neurologic deficits consistent with the vascular area of the brain (16), consistent with NIHSS ≤15, aged 18 years or older at the onset of the event, living in Jakarta during the research process, discharged home, and who spoke Indonesian were included. Patients who could not provide informed consent, those who were released to long-term nursing care, and those with pre-stroke dementia, end-stage cancer, or other medical disorders likely to cause death within 1 year were excluded.

In January 2022, the Faculty of Medicine Ethics Committee at Universitas Indonesia accepted the research protocol, which was given the protocol number KET-4/UN2.F1/ETIK/PPM.00.02/2022. This trial was also registered in the International Standard Randomized Controlled Trial Number (ISRCTN) with trial number ISRCTN11495822. The research adheres to the World Medical Association's Code of Ethics (Declaration of Helsinki) (17). The research was carried out without any deviations from the original protocol. Therefore, no procedure alterations occurred after the trial began. The reporting of this study also refers to and adheres to the Consolidated Standards of Reporting Trials (CONSORT) 2010 Statement (18).

### Intervention

**Figure 1:**
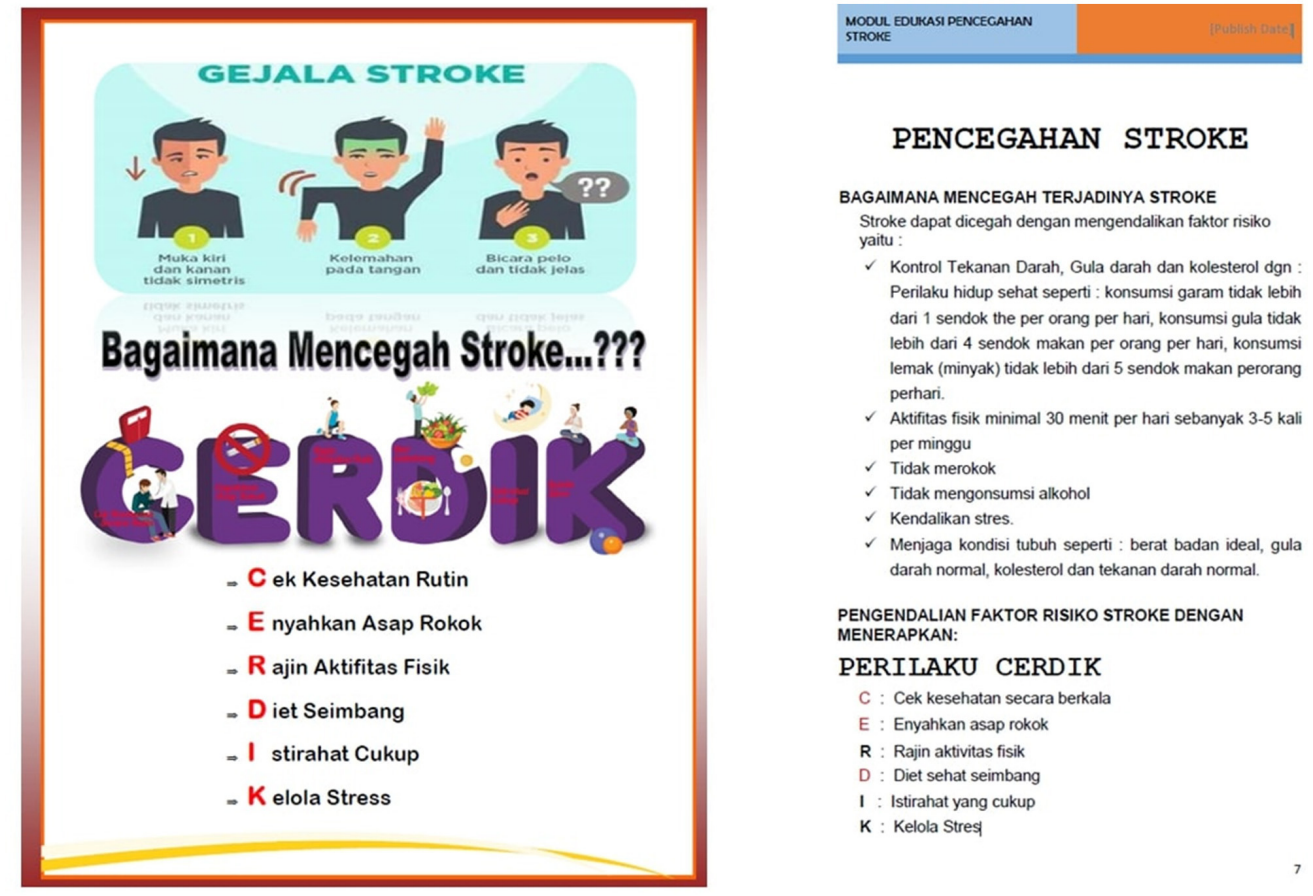
Example of content from the SSE booklet held by the patient **(A)** and educator **(B)**. Examples of the content displayed both discuss stroke prevention.

The intervention group received SSE and standard education (overview of stroke, its causes, medications to take, and home care). SSE uses a visual-based educational model emphasizing concrete examples of self-care and increasing self-efficacy (Figure 1), such as the type of food consumed and recommended exercise schedule (15). In total, one physician and two nurses delivered SSE to each patient and caregiver face-to-face for approximately 90 min before hospital discharge. The physicians and nurses who serve as educators have received prior training in delivering SSE to intervention patients. In total, two booklets serve as educational resources for SSE, which have been developed in our previous research (19). Figure 1 depicts an example of the contents of these two booklets, while the entire contents can be found in the Supplementary material. The first booklet (Figure 1, left) is given to the patient and typically contains more colorful images and diagrams. The second booklet (Figure 1, right) is held by the educator (doctor or nurse) and typically contains more theory regarding the educational material. All booklets used in this study were printed in Indonesian and aimed at patients and health workers who can speak Indonesian. After 1 month and 3 months of discharge, the educator refreshed the booklet for the patient for ~90 min to maintain fidelity. Refreshed content consists of the important elements that have been stated in the booklet (19). The control group did not receive these content refreshments, but they did participate in each brief follow-up session. The intervention and control groups were enrolled from different wards to avoid possible bias due to booklet sharing.

### Study procedures and outcomes

All patients were interviewed in person after their discharge. All participants received a card with the time, place, and contact information for a follow-up visit at the Stroke Subspecialty Cipto Mangunkusumo National Hospital. Blinded research assistants collected self-care, self-efficacy, stroke risk, anthropometric indices, and neurologic assessment data. Doctors and nurses conducted participant interviews and reviewed medical records to collect all data. To account for the Hawthorne Effect, the research team called all participants, including those allocated to standard care 2 weeks following discharge to check on their wellbeing and document outcome events. In addition, in the 1st and 3rd post-discharge months, patients in both groups were required to return to the hospital as per the instructions on their cards.

The primary outcome of this research is the change in self-care, self-efficacy, and stroke risk scores after 1 and 3 months of discharge. The primary outcome was assessed by an independent general practitioner. Changes in self-care and self-efficacy are assessed based on the Hypertension Self-Care Instrument (20), validated in Indonesia (19). As shown in the Supplementary material, the validated instrument has 17 items. Each item consists of a scale of 1 to 4, which the patient answers. To assess self-care, a value of 1 means “never,” 2 means “rarely,” 3 means “often,” and 4 means “always.” Meanwhile, to assess self-efficacy, a value of 1 means “not sure,” 2 means “not sure enough,” 3 means “sure,” and 4 means “very sure.” The total score of the Hypertension Self-Care Instrument is 68, both for self-care and self-efficacy. The greater the value of the Hypertension Self-Care Instrument, the better the patient's self-care and self-efficacy.

Stroke risk factors, meanwhile, were measured using the Feigin Stroke Risk Score (2). The Feigin Stroke Risk Score is widely utilized in Indonesia, including at the center of this trial, and has been validated in Indonesia (19). This stroke risk score is comprised of age score, blood pressure score, blood sugar score, cholesterol score, BMI score, family history of stroke score, alcohol score, smoking score, exercise score, and diet score. Depending on the patient's risk factors, each component (except diet) has a value of 0, 1, 2, or 3, with 0 being the lowest and 3 being the highest. The diet component is evaluated with a 0 or 1 value, where 0 indicates that the patient follows the recommended diet and 1 indicates that they do not. The sum of all component values is the Feigin Stroke Risk Score, which ranges from 0 to 28. A score of 0 indicates a low-risk factor for stroke, while a value of 28 indicates a very high-risk factor for stroke. Each stroke risk factor component in the Feigin Stroke Risk Score was anticipated to improve significantly between 1 month and 3 months following discharge.

The secondary outcome of this study is the functional outcome and blood viscosity at 1 month and 3 months after discharge. Functional outcome was measured by the mRS score (21) and the Barthel index (22), while blood viscosity was measured by the Digital Microcapillary Instrument (23) and expressed in centipoise (cP). There have been no changes to the study results since the trial began.

### Sample size and statistical analysis

Minimal sample estimates were based on our 70-patient pilot study, which revealed a 2.26-point (SD 0.46-point) decrease in the intervention group's stroke risk score compared to the standard care. Thereafter, we conducted a power analysis using a lower 2-point estimate, a two-sided significance threshold of 5%, and 80% power. Given a dropout rate of 10%, each group required 54 participants. An independent sample *t*-test compared trial arms' mean stroke risk reduction, self-care score, and self-efficacy score. An independent sample *t*-test compared all secondary outcomes between study arms. Before analysis, Microsoft Excel was used to input data gathering into a main table (Microsoft Corp, Redmond, WA, USA). Statistical Package for the Social Sciences (SPSS) 20 was used to analyze and display tabulated data (IBM Corp, Armonk, NY, USA). All categorical variables are reported as frequencies and analyzed using the chi-square test, while all numerical variables are written as means (standard deviation) and analyzed using an independent sample *t*-test. For both primary and secondary outcomes, a *p*-value <0.05 is considered to be statistically significant.

## Results

**Figure 2:**
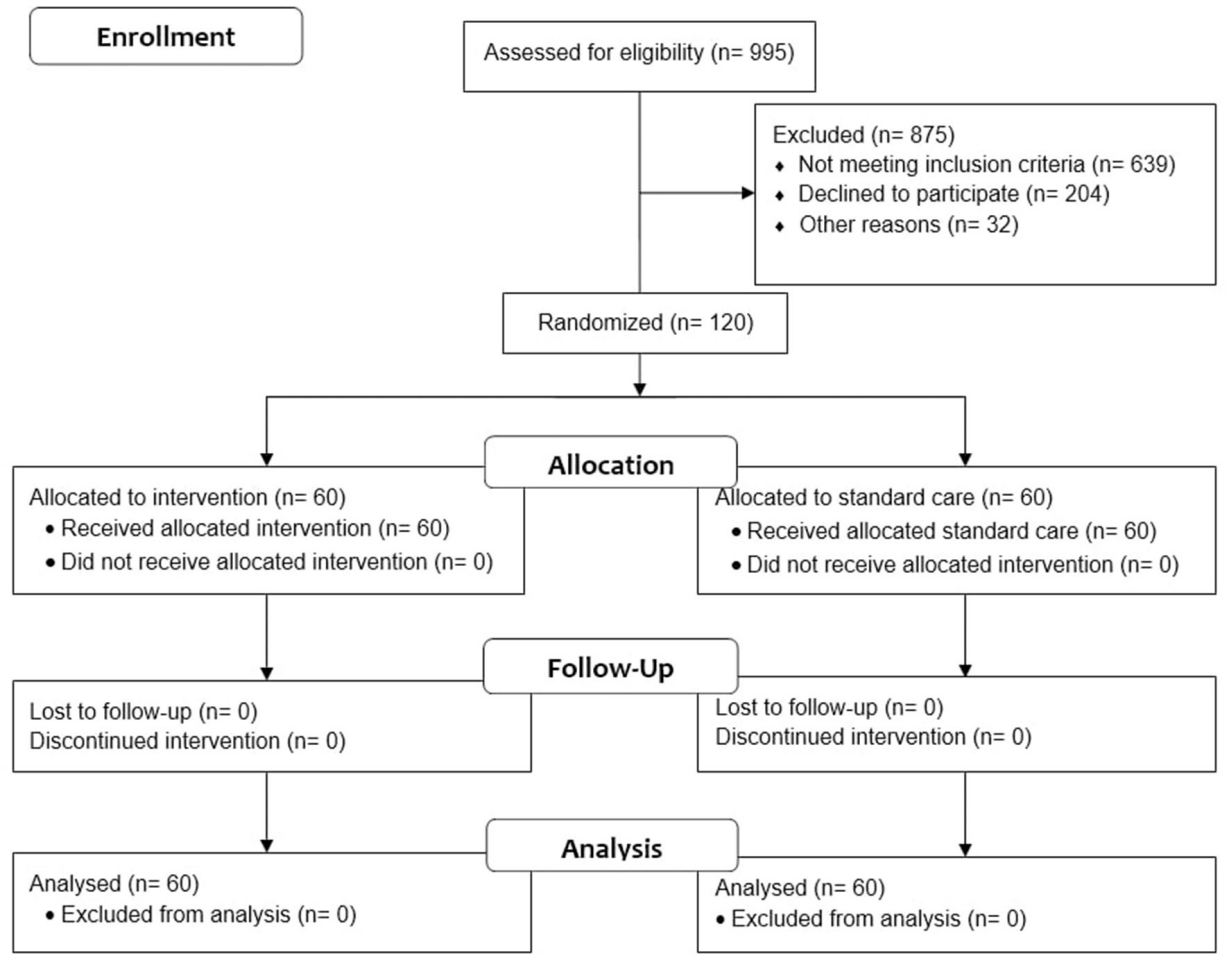
CONSORT flow diagram of the self-efficacy and self-care-based stroke care model for risk factor modification study (18).

As indicated in Figure 2, 995 individuals were screened, and 120 (12%) were qualified for participation. Between January 2022 and October 2022, we assessed the population for non-specific symptoms associated with mild-to-moderate ischemic stroke. Patients were observed at baseline (during hospitalization), 1 month, and 3 months following discharge. During the trial, there was neither loss to follow-up patients nor protocol deviation, so the total patients analyzed matched those who were enrolled. The trial was stopped after the enrolled samples met the minimum sample size. All randomized participants were included in the intention-to-treat analysis.

Baseline demographic and clinical characteristics were well-balanced between the two groups, as shown in Table 1. The gender distribution in the two trial arms was similar, accompanied by an almost similar age distribution. Demographically, the study subjects were dominated by Sundanese in the intervention group and Javanese in the standard care group. The two trial arms show a more dominant distribution in subjects with junior high school education or below, urban, unmarried, employed, cared for by their children, and family as the primary social environment. The average NIHSS score suggested that the severity of strokes in this patient group was moderate. The patients' main comorbidities were hypertension and hyperlipidemia, with a high stroke risk score.

**Table 1:** Baseline demographic and clinical characteristics of study participants.

**Characteristics**	**Intervention (*n* = 60)**	**Standard care (*n* = 60)**	***p*-value**
**Gender**			
Male	27 (45.0%)	30 (50.0%)	0.715
Female	33 (55.0%)	30 (50.0%)	
Age (years)	57.56 (13.34)	57.3 (14.04)	0.107
**Ethnicity**			
Javanese	21 (35.0%)	31 (51.7%)	0.127
Sundanese	24 (40.0%)	15 (25.0%)	
Bataknese	4 (6.7%)	7 (11.7%)	
Others	11 (18.3%)	7 (11.7%)	
**Locality**			
Urban	34 (56.7%)	32 (53.3%)	0.854
Rural	26 (43.3%)	28 (46.7%)	
**Education**			
Junior high school or below	12 (20.0%)	19 (31.7%)	0.211
Senior high school or above	48 (80.0%)	41 (68.3%)	
**Marital status**			
Married	6 (10.0%)	3 (5.0%)	0.488
Unmarried	54 (90.0%)	57 (95.0%)	
**Employment**			
Employed	44 (73.3%)	38 (63.3%)	0.326
Unemployed	16 (26.7%)	22 (36.7%)	
**Caregiver**			
Parent	5 (8.3%)	3 (5.0%)	0.635
Siblings	6 (10.0%)	9 (15.0%)	
Spouse	10 (16.7%)	13 (21.7%)	
Children	39 (65.0%)	35 (58.3%)	
**Social environment**			
Family	41 (68.3%)	38 (63.3%)	0.061
Friends	8 (13.3%)	17 (28.3%)	
Neighbors	11 (18.3%)	5 (8.3%)	
Hospital length of stay	6.8 (2.25)	7.1 (2.02)	0.395
NIHSS admission	6.13 (2.47)	6.15 (1.86)	0.967
NIHSS discharged	1.95 (1.36)	2.07 (1.19)	0.618
Blood viscosity (cP)	6.55 (1.25)	6.41 (1.75)	0.609
Barthel index	53.75 (27.65)	50.16 (26.87)	0.473
mRS	2.53 (1.40)	2.70 (1.43)	0.521
**Comorbidity**			
Diabetes (yes)	18 (30.0%)	18 (30.0%)	1.000
Hypertension (yes)	59 (98.3%)	60 (100%)	0.315
CKD (yes)	2 (3.3%)	6 (10.0%)	0.135
CAD (yes)	4 (6.7%)	2 (3.3%)	0.402
Hyperlipidemia (yes)	47 (78.3%)	49 (81.7%)	0.648
Stroke risk score	14.77 (2.76)	13.67 (3.16)	0.854
Age score	1.30 (0.87)	1.26 (0.92)	0.839
Blood pressure score	1.37 (0.64)	1.35 (0.66)	0.888
Blood sugar score	1.85 (0.99)	1.75 (1.07)	0.595
Cholesterol score	1.50 (0.95)	1.45 (0.99)	0.779
BMI score	1.16 (1.39)	1.16 (1.43)	1.000
Family stroke history score	0.7 (0.81)	0.73 (0.69)	0.808
Alcohol score	1.67 (1.24)	1.70 (1.28)	0.885
Smoking score	2.00 (0.74)	2.01 (0.99)	0.917
Activity score	2.33 (0.68)	2.41 (0.81)	0.543
Diet score	0.88 (0.33)	0.82 (0.39)	0.311
Self-care score	23.32 (5.62)	23.47 (6.64)	0.894
Self-efficacy score	25.18 (9.46)	25.33 (10.10)	0.933

^*^p-value <0.05 is considered statistically significant.

Data are means (SD) or numbers (%).

BMI, body mass index; cP, centipoise; mRS, modified Rankin Scale; NIHSS, National Institutes of Health Stroke Scale.

**Figure 3:**
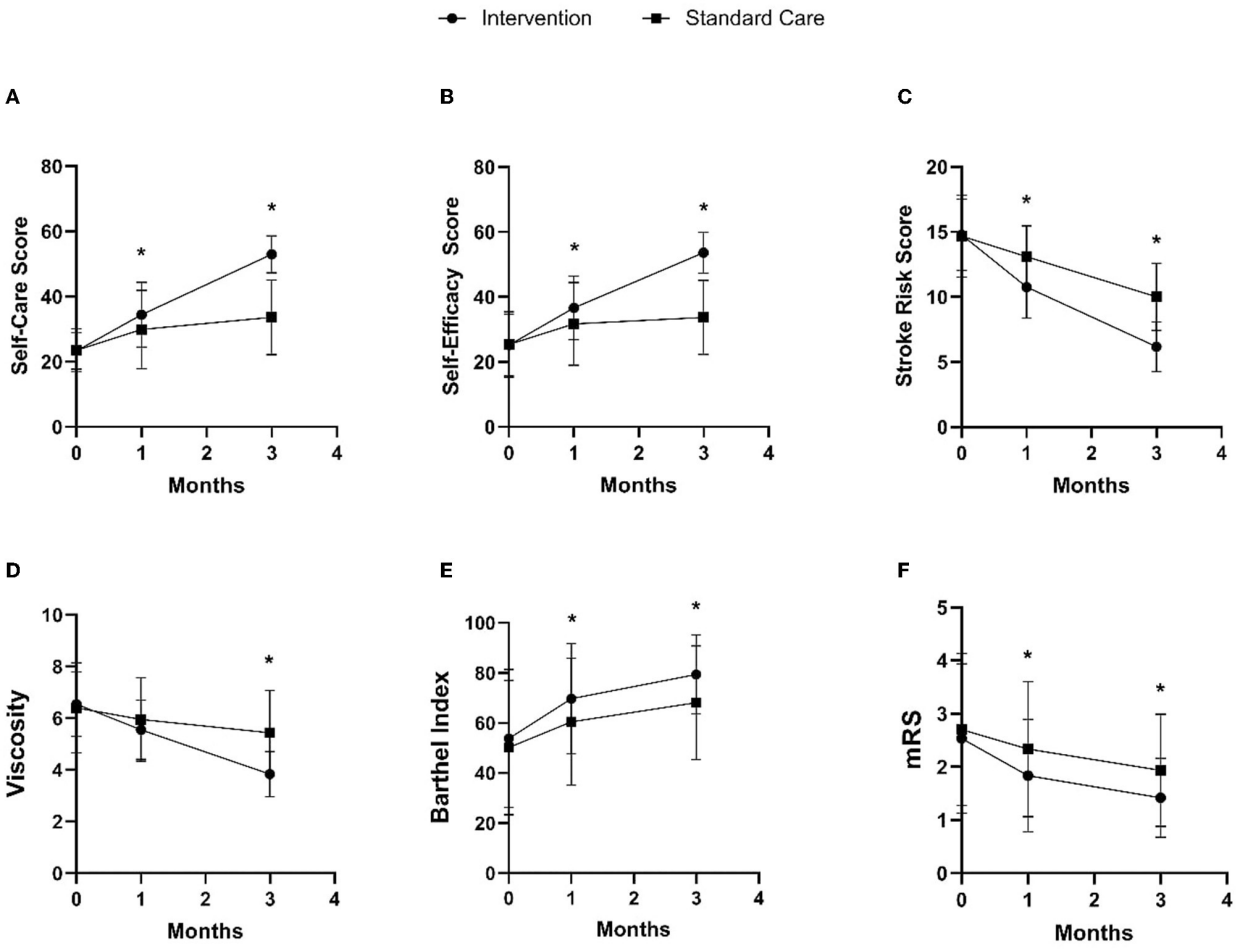
Comparison of intervention and standard care group on changes in self-care **(A)**, self-efficacy **(B)**, stroke risk **(C)**, blood viscosity **(D)**, Barthel index **(E)**, and mRS **(F)** in the 1st and 3rd months after discharge. *p-value < 0.05.

The intervention group showed changes in self-care, self-efficacy, and stroke risk compared to the standard care group, as shown in Figures 3A–C and Table 2. In the 1st month after discharge, the intervention group showed differences in increasing self-care by 4.56 points (95% CI: 0.57, 8.56), increasing self-efficacy by 4.95 points (95% CI: 0.84, 9.06), and a significant reduction in stroke risk by 2.33 points (95% CI: −3.19, −1.47) compared to the standard care group. In the 3rd month after discharge, the intervention group also increased self-care by 19.28 points (95% CI: 16.01, 22.56), increased self-efficacy by 19.95 points (95% CI: 16.61, 23.28), and had a significant reduction in stroke risk by 3.83 points (95% CI: −4.65, −3.01) compared to the standard care group.

**Table 2:** Comparison of intervention and standard care group on changes in the 1st and 3rd months after discharge.

**Parameters**	**1-month follow-up**			**3-month follow-up**		
	**Intervention (*****n*** = **60)**	**Standard care (** ***n*** **-60)**	**Mean difference (95%CI)**	**Intervention (*****n*** = **60)**	**Standard care (*****n*** = **60)**	**Mean difference (95%CI)**
Self-care score	34.43 (9.95)	29.87 (12.06)	4.56(0.57, 8.56)	52.93 (5.72)	33.65 (11.49)	19.28 (16.01, 22.56)
Self-efficacy score	36.65 (9.80)	31.70 (12.76)	4.95 (0.84, 9.06)	53.63 (6.29)	33.68 (11.31)	19.95 (16.61, 23.28)
Stroke risk score	10.75 (2.38)	13.08 (2.37)	−2.33 (−3.19, −1.47)	6.17 (1.91)	10.00 (2.59)	−3.83 (−4.65, −3.01)
Blood viscosity (cP)	5.55 (1.15)	5.95 (1.61)	−0.40 (−0.91, −0.10)	3.84 (0.88)	5.44 (1.64)	−1.60 (−2.07, −1.12)
Barthel index	69.67 (22.11)	60.42 (25.35)	9.25 (0.65, 17.85)	79.33 (15.82)	68.08 (22.68)	11.25 (4.17, 18.32)
mRS	1.83 (1.06)	2.33 (1.27)	−0.50 (−0.92, −0.08)	1.42 (0.74)	1.93 (1.06)	−0.52 (−0.85, −0.19)
Blood pressure score	1.08 (0.33)	1.28 (0.52)	−0.20 (−0.36, −0.04)	0.58 (0.49)	1.02 (0.29)	−0.44 (−0.58, −0.29)
Blood sugar score	1.10 (0.71)	1.55 (0.81)	−0.45 (−0.72, −0.18)	0.42 (0.49)	0.73 (0.71)	−0.32 (−0.54, −0.09)
Cholesterol score	1.03 (0.76)	1.22 (0.90)	−0.18 (−0.48, −0.12)	0.38 (0.49)	1.08 (0.74)	−0.70 (−0.93, −0.47)
BMI score	0.85 (0.89)	1.10 (1.17)	−0.25 (−0.63, −0.13)	0.57 (0.87)	1.02 (1.08)	−0.45 (−0.80, −0.09)
Alcohol score	1.08 (0.85)	1.36 (1.07)	−0.28 (−0.63, −0.07)	0.38 (0.49)	0.98 (0.95)	−0.60 (−0.87, −0.32)
Smoking score	1.62 (0.83)	1.90 (0.93)	−0.28 (−0.60, −0.04)	0.78 (0.64)	1.17 (1.01)	−0.38 (−0.69, 0.08)
Activity score	1.77 (0.53)	2.23 (0.79)	−0.47 (−0.71, −0.22)	0.98 (0.65)	1.68 (0.93)	−0.70 (−0.99, −0.41)
Diet score	0.22 (0.42)	0.43 (0.49)	−0.20 (−0.38, −0.05)	0.067 (0.25)	0.37 (0.49)	−0.30 (−0.44, −0.16)

^*^Data are means (SD).

BMI, body mass index; CI, confidence interval; cP, centipoise; mRS, modified Rankin Scale, SD, standard deviation.

In addition to the main result, the intervention group demonstrated changes in clinical and functional markers, including blood viscosity, Barthel index, and mRS, compared to the standard care group, as shown in Figures 3D–F and Table 2. In the 1st month after discharge, the intervention group showed an increase in Barthel index by 9.25 points (95% CI: 0.65, 17.85) and an improvement in mRS by 0.50 points (95% CI: −0.92, −0.08) compared to the standard care group. In the 3rd month after discharge, the intervention group also showed a decrease in blood viscosity by 1.60 cP (95% CI: −2.07, −1.12), an increase in Barthel index by 11.25 points (95% CI: 4.18, 18.32), and mRS improvement by 0.51 points (95% CI: −8.47, −0.19) compared to the standard care group.

**Figure 4:**
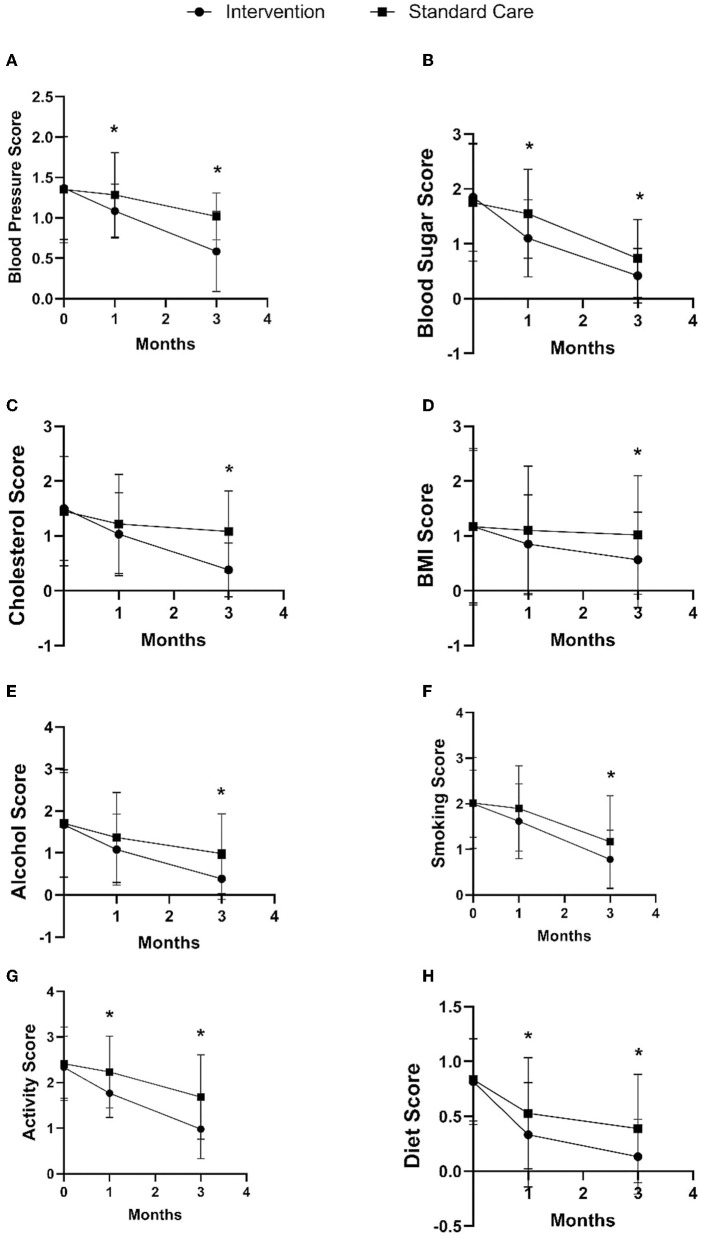
Comparison of intervention and standard care group on changes in blood pressure **(A)**, blood sugar **(B)**, cholesterol **(C)**, BMI **(D)**, alcohol **(E)**, smoking **(F)**, activity **(G)**, and diet **(H)** in the 1st and 3rd months after discharge. *p-value < 0.05.

Furthermore, the intervention group also showed changes in the modifiable risk factor component in the stroke risk score compared to the standard care group, as shown in Figure 4 and Table 2. In the 1st month after discharge, the intervention group showed a decrease in blood pressure by 0.20 points (95% CI: −0.36, −0.04), decreased blood sugar by 0.45 points (95% CI: −0.72, −0.18), increased activity by 0.46 points (95 % CI: −0.71, −0.22), and significantly improved diet by 0.20 points (95% CI: −0.38, −0.05) compared to the standard care group. In the 3rd month after discharge, the intervention group showed decreased blood pressure by 0.44 points (95% CI: −0.58, −0.28), decreased blood sugar by 0.31 points (95% CI: −0.54, −0.09), decreased cholesterol by 0.70 points (95% CI: −0.93, −0.47), decreased BMI by 0.45 points (95% CI: −0.80, −0.09), decreased alcohol consumption by 0.60 points (95% CI: −0.87, −0.33), decreased smoking by 0.39 points (95% CI: −0.68, −0.08), increased physical activity by 0.70 points (95% CI: −0.99, −0.41), and improved diet by 0.26 points (95% CI: −0.44, −0.16) as compared to the standard care group.

## Discussion

The SCORES trial focuses on conducting SSE to modify stroke risk factors and was the first RCT to evaluate the efficacy between the two. When followed up after 3 months, SSE significantly increased patient self-efficacy and self-care by 19.95 points and 19.28 points, respectively. A similar increase between self-efficacy and self-care implicitly shows that self-care and self-efficacy are closely related and mutually reinforcing. Engaging in self-care activities, such as exercise, proper nutrition, and stress management techniques, can improve physical and cognitive functioning in stroke patients (24, 25). This improvement in functioning can, in turn, increase self-efficacy as individuals can perform daily tasks and activities more effectively (26). Additionally, self-care practices can help stroke patients develop a sense of control over their recovery and wellbeing, which can further boost self-efficacy (27). Conversely, increased self-efficacy can also improve stroke patients' self-care practices. Individuals may be more inclined to participate in self-care activities, such as sticking to a balanced diet or exercising consistently, as their confidence in their skills increases (28). Furthermore, self-efficacy may also play a role in maintaining self-care practices, as individuals who believe they can successfully manage their health are more likely to continue to engage in self-care behaviors over time (29). Therefore, it is essential to consider self-care and self-efficacy in the rehabilitation and recovery of stroke patients.

SSE can also reduce the patient's stroke risk by modifying their risk factors. This change occurs in each component of the Feigin Stroke Risk Score: age, blood pressure, blood sugar, cholesterol, BMI, family history of stroke, alcohol, smoking, exercise, and diet. This change occurs because SSE explicitly relates to each risk factor by defining concrete steps in the booklet that can be taken home. Each of these risk factors is also related to one another. Physical activity, for example, can help lower blood pressure and improve cardiovascular health, while a healthy diet can help control blood sugar and improve cholesterol levels (30, 31). However, self-care knowledge cannot be separated from the importance of self-efficacy in bridging the knowing-doing gap (7). Individuals who feel they can effectively manage their health are more likely to participate in self-care behaviors if they have high self-efficacy (7). This is evidenced by the more considerable mean difference in the 3rd month compared to the first.

As a result of the risk factor modification, this trial also showed changes in functionality, namely improvements in the mRS and Barthel Index, and microcirculation, namely a decrease in blood viscosity. Self-care practices such as physical activity, proper nutrition, and stress management can help to improve physical and cognitive functioning in stroke patients. This improvement in functioning can increase the patient's ability to perform activities of daily living and improve their level of independence, reflected in a better score in the mRS and Barthel index. A healthy diet low in saturated fats and cholesterol can also help lower blood viscosity by reducing the amount of lipids in the blood. As previously explained, self-efficacy can also help to promote the maintenance of self-care practices over time, as individuals who believe they can successfully manage their health are more likely to continue to engage in self-care behaviors that lower blood viscosity.

This study has strengths and limitations. The strength of this study is that it is the first RCT to examine the impact between SSE and changes in stroke risk factors and their impact on functional outcomes and blood viscosity. In addition, since the intervention was developed for both sexes, all ages, and other basic demographic features, it may be consistently and successfully administered by other clinicians in various contexts. Conversely, because it was carried out at a national central hospital, many patients have complex strokes, so the enrollment process was challenging. Although this limitation has been overcome by calculating the minimum sample size, further research with a larger sample size is highly desirable. The number of patients studied was modest, so the results need to be confirmed in a larger sample size and diverse stroke patient populations. Lastly, this research also has limitations in terms of infrastructure, finance, and logistics for bettering stroke prevention. However, we view this limitation as an advantage as the educational approach can demonstrate a positive outcome for stroke prevention under limited conditions.

## Conclusion

SSE can increase self-care and self-efficacy scores, modify risk factors, improve functional outcomes, and reduce blood viscosity. Therefore, it is essential to consider self-care and self-efficacy in the rehabilitation and recovery of stroke patients.

## Data availability statement

The original contributions presented in the study are included in the article/Supplementary material, further inquiries can be directed to the corresponding author.

## Ethics statement

The studies involving human participants were reviewed and approved by Universitas Indonesia Faculty of Medicine Ethics Committee. The patients/participants provided their written informed consent to participate in this study.

## Author contributions

AR, UP, EW, and MF: conceptualization. AR and UP: methodology. EW: software, writing—review and editing and project administration. AR and SH: validation. AR and EW: formal analysis, data curation, and writing—original draft preparation. AR, UP, SA, and EW: investigation. AR: resources and funding acquisition. AR, SH, and MF: supervision. All authors: visualization. The published version of the article has been reviewed and approved by all the authors.
