# Supplementary material for: Exploring the self-efficacy and self-care-based stroke care model for risk factor modification in mild-to-moderate stroke patients
Source: Front Neurol. 2023 May 11;14:1177083. doi: 10.3389/fneur.2023.1177083 (PMC10213644; doi:10.3389/fneur.2023.1177083)
Supplement: Supplementary file 1 [file Data_Sheet_1.DOCX]

| Question |
| --- |
|  |
|  |
| 1. Do regular physical activity (30 minutes of walking 4-5 times/week) |
| 1. Read nutrition labels to check the salt content of the food you buy |
| 1. Replacing instant food with traditional homemade food |
| 1. Limiting the use of high-salt condiments (e.g., ketchup) |
| 1. Eat less than 1 teaspoon of salt per day (6 g) |
| 1. Eat fewer foods high in saturated fat (e.g., red meat, butter) and trans-fat (e.g., white butter) |
| 1. Use broil, bake, or steam instead of frying when cooking |
| 1. Read the nutrition label of the food you buy to see the saturated fat and trans-fat content |
| 1. Replacing high-fat foods (e.g., fried chicken) with low-fat foods (e.g., grilled chicken) |
| 1. Limit total calories from fat each day (less than 65 grams) |
| 1. Eat fruits and vegetables every day (5-6 servings) |
| 1. Get used to not smoking |
| 1. Take medication for blood pressure/blood sugar/cholesterol |
| 1. Maintain weight |
| 1. Monitor situations that can cause high levels of stress that can lead to an increase in blood pressure |
| 1. Participate in activities that reduce stress (exercise, meditation) |
| 1. Carry out regular health checks |

**Supplement 1.** Hypertension Self-Care Instrument which has been validated in the study population.
