# Supplementary material for: Exploring the self-efficacy and self-care-based stroke care model for risk factor modification in mild-to-moderate stroke patients
Source: Front Neurol. 2023 May 11;14:1177083. doi: 10.3389/fneur.2023.1177083 (PMC10213644; doi:10.3389/fneur.2023.1177083)
Supplement: Supplementary file 2 [file Image_1.PDF]

Booklet Edukasi Pencegahan Stroke Untuk Pasien dan Keluarga

# AYO KITA CEGAH...!!!

STROKE

Oleh:

UKE PEMILA

#modeledukasipencegahanstroke#mila#

Stroke

Adalah gangguan peredaran darah ke otak akibat sumbatan atau pecahnya pembuluh darah otak yang dapat mengakibatkan gangguan pada otak bahkan kematian.

Penyebab Stroke

Cegah Faktor Risiko :

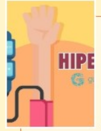
Hipertensi

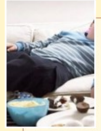
Aktifitas fisik yang kurang

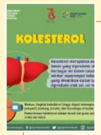

Kolesterol

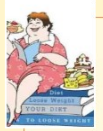
Diet yang tidak tepat

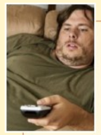
Kegemukan

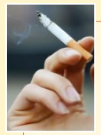
Merokok

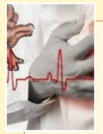
Penyakit jantung

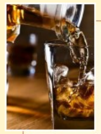
Konsumsi alkohol

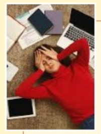
Stress

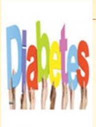

Diabetes

Bahaya Stroke

Kematian → Kecacatan

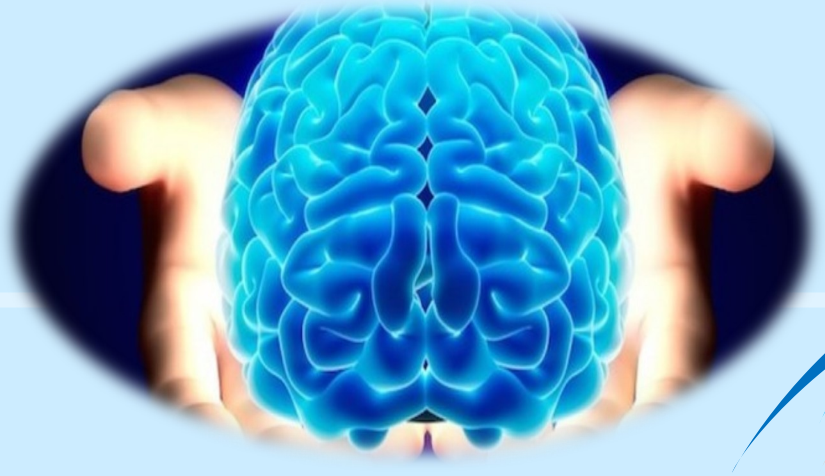

Hidup Tenang

Tanpa Risiko Stroke

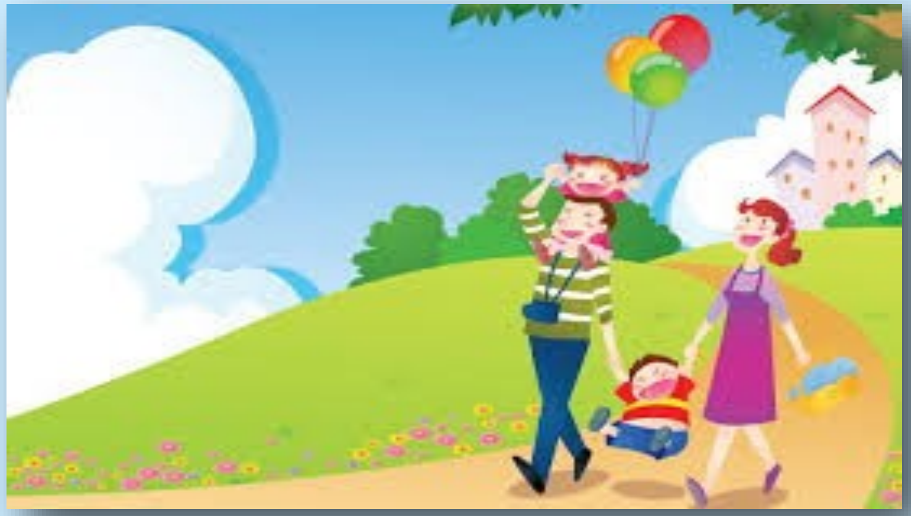

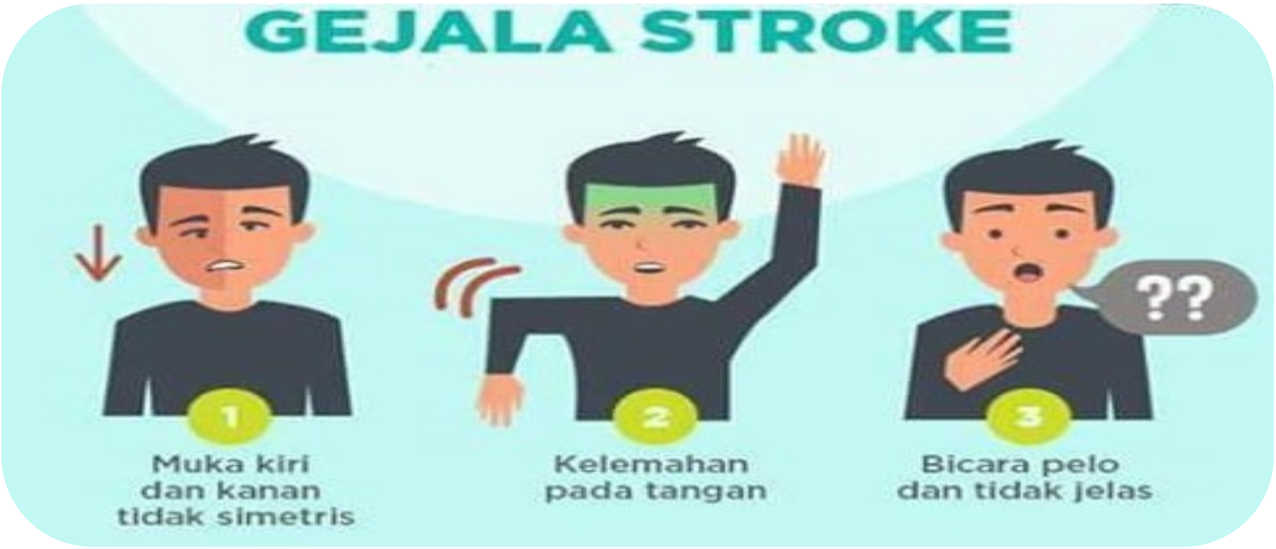

Bagaimana Mencegah Stroke...???

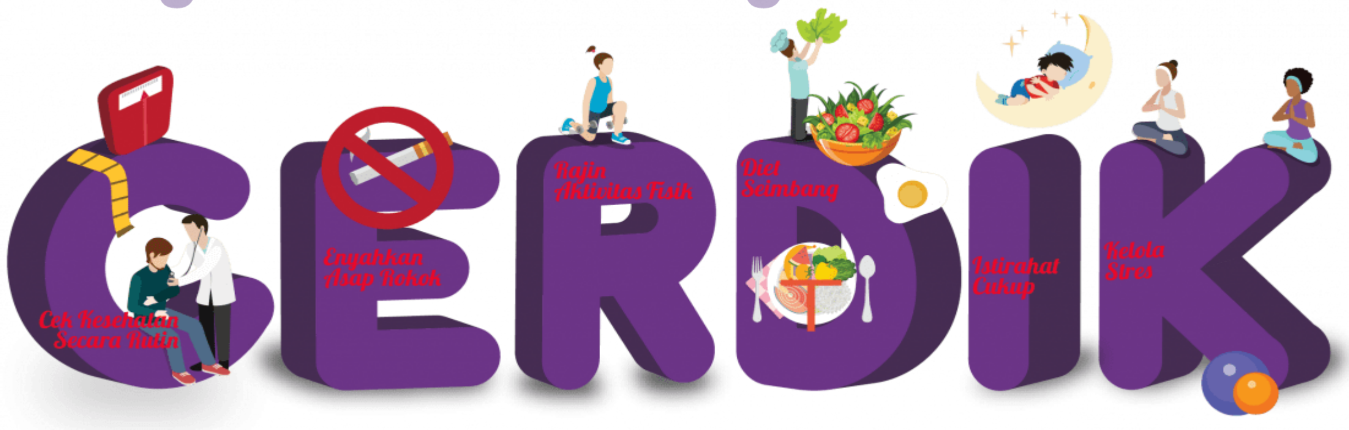

- ⇒ **C** ek Kesehatan Rutin
- ⇒ **E** nyahkan Asap Rokok
- ⇒ **R** ajin Aktifitas Fisik
- ⇒ **D** iet Seimbang
- ⇒ **I** stirahat Cukup
- ⇒ **K** elola Stress

Faktor Risiko Stroke

#1

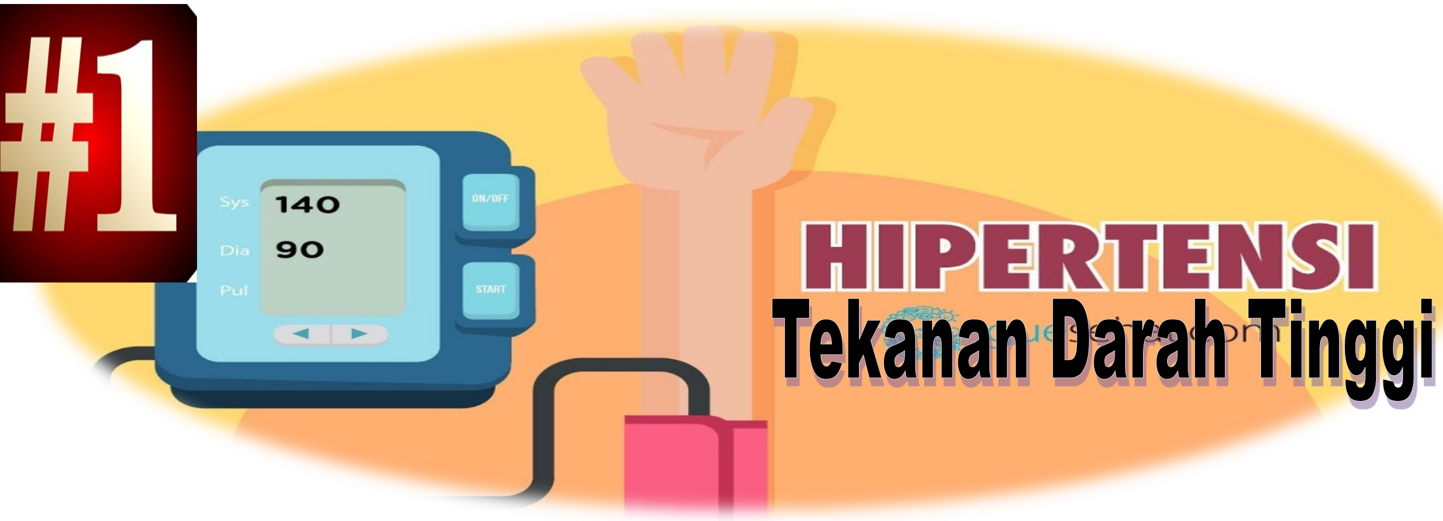

HIPERTENSI
Tekanan Darah Tinggi

adalah suatu keadaan dimana tekanan darah sistolik ≥ 140 mmHg dan atau tekanan diastolik ≥ 90 mmmHg

Faktor Risiko

A. Faktor risiko yang tidak dapat diubah , yaitu : Umur, jenis kelamin dan riwayat keluarga

B. Faktor risiko yang dapat diubah, yaitu : Konsumsi garam berlebih, kurang aktifitas fisik, kegemukan, merokok, kurang makan sayur dan buah, dislipidemia dan stress.

Gejala

- ⇒ Sakit Kepala
- ⇒ Jantung Berdebar debar
- ⇒ Pusing
- ⇒ Gelisah
- ⇒ Rasa Sakit di dada
- ⇒ Penglihatan Kabur
- ⇒ Mudah Lelah

Komplikasi :

1. Gangguan jantung
2. Gangguan saraf dan otak
3. Gangguan fungsi ginjal
4. Gangguan Penglihatan

Mengendalikan

1 • Gaya hidup sehat

2 • Minum obat teratur

9 KELOLA STRES

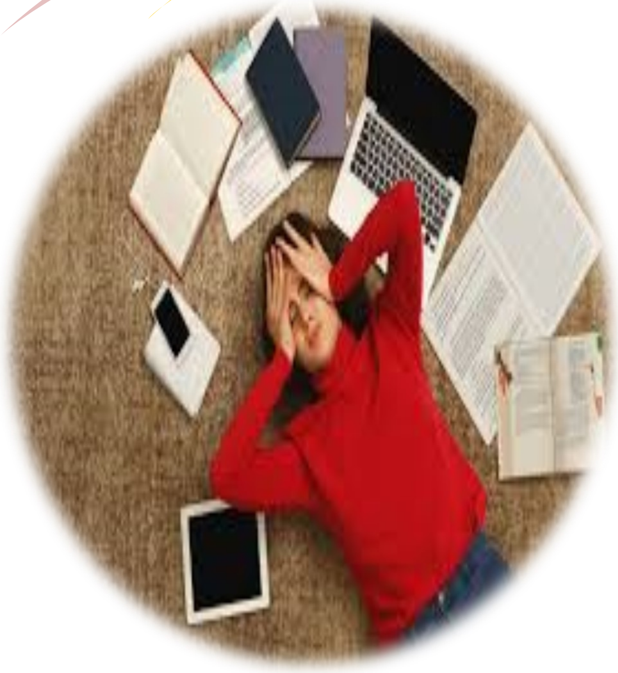

Gejala Stres

- ⇒ Gelisah
- ⇒ Muka Pucat
- ⇒ Sulit Tidur
- ⇒ Nafsu makan berkurang/berlebih
- ⇒ Mudah tersinggung
- ⇒ Sulit Kosentrasi
- ⇒ Sakit Kepala
- ⇒ Sakit Maag

Mengelola Stres

1. Bicarakan keluhan dgn seseorang yang dipercaya
2. Melakukan kegiatan sesuai minat dan kemampuan
3. Jaga kesehatan dgn tidur cukup, makan bergizi seimbang, olahraga/ aktifitas fisik secara teratur dan berperilaku hidup bersih dan sehat
4. Kembangkan hobi yang bermanfaat
5. Meningkatkan ibadah dan mendekatkan diri pada Tuhan
6. Berfikir positif
7. Tenangkan pikiran dengan relaksasi

Teknik Relaksasi

A. Duduk dengan posisi santai dan nyaman. Bayangkan hal yang meneyenangkan dengan mata terpejam

B. Tarik nafas dari hidung, tahan 3 hitungan lalu hembuskan nafas dari mulut. Bayangkan seolah beban pikiran dilepaskan. Ulangi sebanyak 3 kali

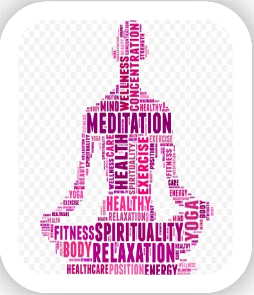

C. Mensyukuri nikmat dari Tuhan YME, merasa ikhlas dan sabar.

8 Konsumsi Alkohol

Bahaya Alkohol

1. Menyebabkan gangguan penurunan fungsi seperti penilaian yang buruk.
2. Reaksi pengambilan keputusan lama
3. Kewaspadaan yang rendah
4. Penurunan tajam penglihatan

Dampak Alkohol bagi kesehatan tubuh

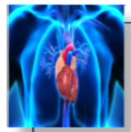

Melemahkan otot jantung dan meningkatkan risiko serangan jantung, stroke dan hipertensi.

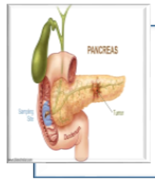

Peradangan pankreas

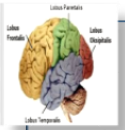

Kerusakan otak

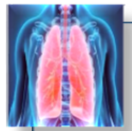

Infeksi paru-paru

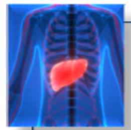

Kerusakan hati

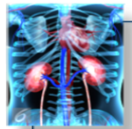

Kerusakan ginjal

2 Diabetes

Adalah penyakit menahun yang ditandai oleh kadar glukosa darah yang melebihi nilai normal.

Biasa dikenal dengan kencing manis.

Batas Normal Kadar Gula Darah Sewaktu adalah < 200 mg/dL
Batas Normal Kadar Gula Darah Puasa adalah < 126 mg/dL

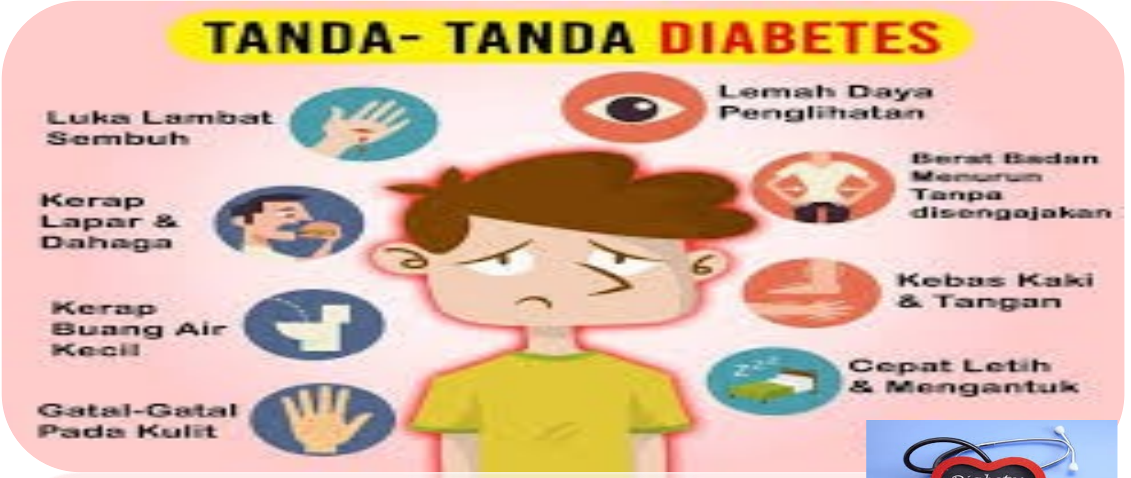

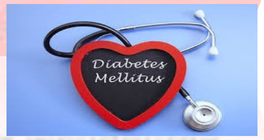

Faktor risiko Diabetes :

Faktor risiko yang tidak dapat diubah :

Umur, jenis kelamin dan riwayat keluarga.

Faktor risiko yang dapat diubah :

Kegemukan, hipertensi, kolesterol, kurang aktifitas

Apa yang harus dilakukan bila terdiagnosa DM...???

1. Mengikuti Edukasi DM
2. Mengatur pola makan
3. Melakukan latihan fisik
4. Mengkonsumsi obat secara teratur
5. Monitoring kadar gula darah

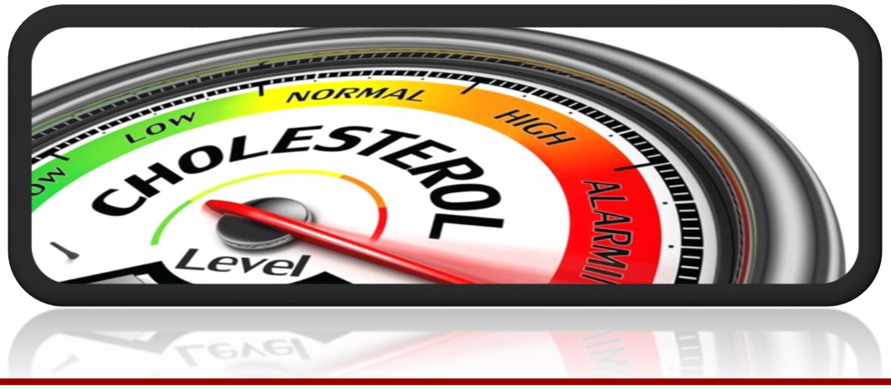

Stroke meningkat pada pasien dengan kadar kolesterol di-atas 240 mg% , setiap kenaikan 38,7 mg% menaikkan ang-ka stroke 25%.

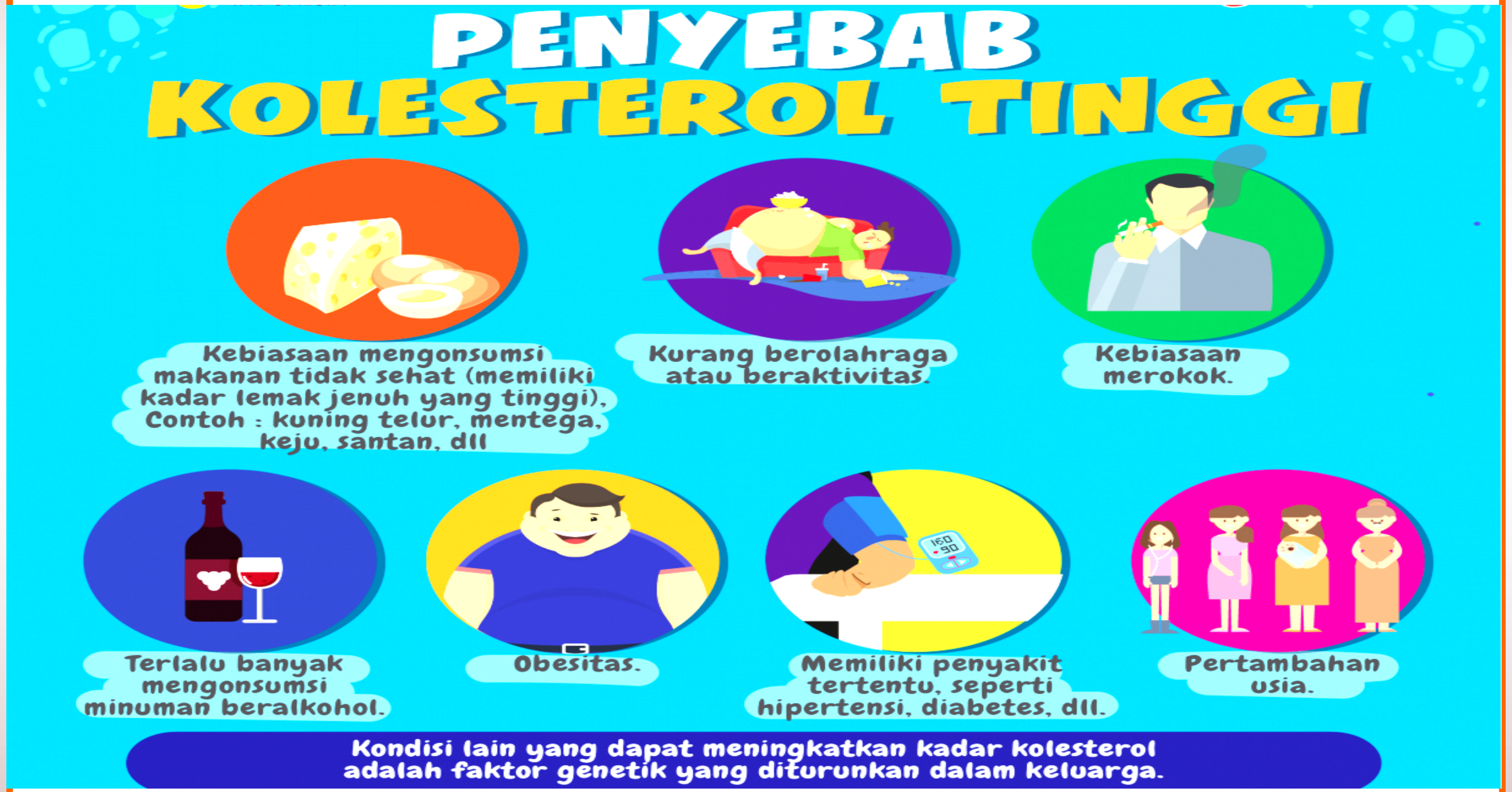

PENCEGAHAN KOLESTEROL TINGGI

- Menerapkan Pola Makan Sehat
- Berolahraga secara teratur
- Menurunkan Berat Badan Berlebih
- Menghentikan Kebiasaan Merokok

MANFAAT BERHENTI MEROKOK

Memberikan peluang lebih besar dalam menga-lokasikan uang atau penghasilan untuk menye-diakan makanan bergizi bagi keluarga, pemenu-han kebutuhan hidup sehari-hari

BERHENTI MEROKOK	MANFAAT
20 menit	• Tekanan darah, denyut jantung, dan aliran darah tepi membaik
12 jam	• Hampir semua nikotin dalam tubuh sudah dimetabo-lisme
	• Kadar CO di dalam darah kembali normal
24 - 48 jam	• Nikotin mulai tereliminasi dari tubuh
	• Fungsi pengecap dan penciuman mulai membaik
	• Sistem kardiovaskular meningkat baik
5 hari	• Fungsi pengecap dan penciuman menjadi lebih mem-baik
2-6 minggu	• Risiko infeksi pada luka setelah operasi berkurang sig-nifikan
	• Fungsi silia pada saluran napas dan fungsi paru mem-baik
	• Napas lebih lega dan batuk-batuk berkurang
1 tahun	• Risiko penyakit jantung koroner menurun
5 tahun	• Risiko stroke menurun seperti orang tidak pernah mero-kok
10 tahun	• Risiko kanker paru berkurang setengahnya
15 tahun	• Semua penyebab kematian dan risiko penyakit jantung menurun selevel dgn orang yang tidak pernah merokok

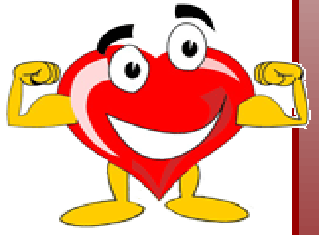

7 MEROKOK

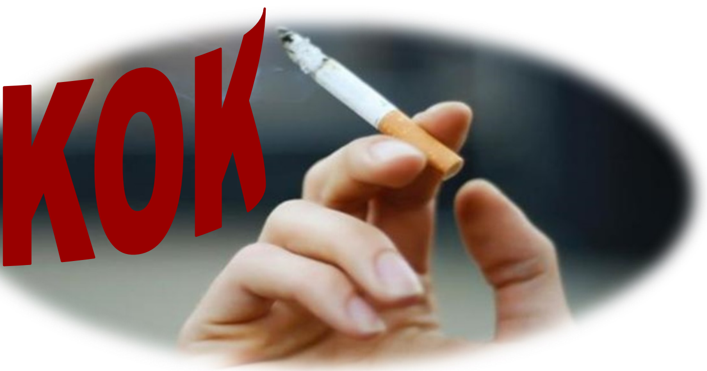

Bahaya Merokok kesehatan individu dibedakan atas :

1. Perokok aktif yakni perokok itu sendiri
2. Perokok pasif yakni orang lain yang tidak merokok namun menghirup asap rokok.

Merokok dapat menyebabkan :

A. Tekanan darah tinggi, penyakit jantung, stroke
B. Asma, penyakit paru kronik
C. Tukak lambung, radang usus besar
D. Kanker paru, kanker payudara, kanker lambung dan kanker usus
E. Mandul/Infertilitas, impotensi, gangguan pada kehamilan dan janin.
F. Cacat bawaan

Menciptakan rumah tanpa Asap Rokok

- Membuat kesepakatan keluarga untuk menciptakan rumah tangga tanpa asap rokok.
- Menegur anggota keluarga yang merokok di dalam rumah.
- Tidak memberi dukungan kepada orang yang merokok dalam bentuk apapun, antara lain dengan tidak memberikan uang untuk membeli rokok, tidak memberikan kesempatan untuk merokok di dalam rumah, tidak menyediakan asbak.
- Tidak menyuruh anak membelikan rokok.
- Orang tua menjadi panutan dalam perilaku tidak merokok
- Melarang anak merokok

4 Aktivitas Fisik yang kurang

Aktivitas fisik merupakan setiap gerakan tubuh yang dapat meningkatkan pengeluaran tenaga atau energi. Aktivitas fisik dapat dilakukan di berbagai situasi dan tempat

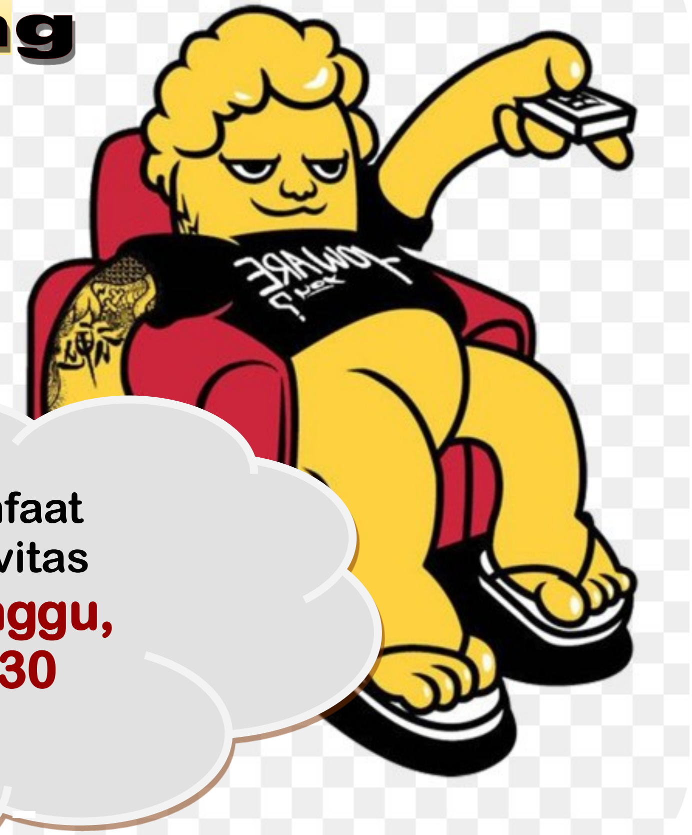

Agar mendapatkan manfaat kesehatan, lakukan aktivitas fisik **150 menit per minggu, idealnya dilakukan 30 menit per hari**

Manfaat aktivitas fisik:

- Menjaga tubuh dalam kondisi yang optimal untuk menjalankan aktivitas keseharian.
- Mengurangi keluhan yang timbul akibat terlalu banyak duduk, berdiri terlalu lama atau bekerja pada posisi yang sama untuk waktu lama.
- Mencegah kegemukan, penyakit jantung pembuluh darah, stroke, diabetes dan penyakit tidak menular lain.
- Menjaga kondisi tubuh tetap bugar dan sehat.
- Menjaga tekanan darah tetap stabil da-

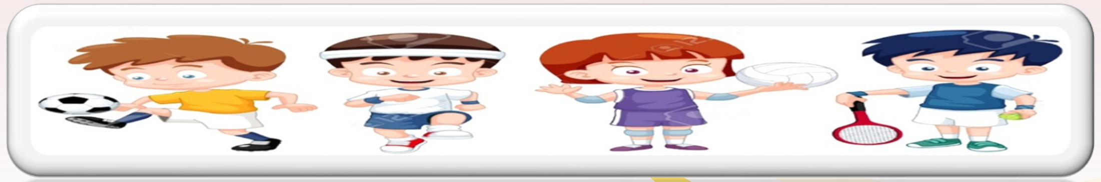

Contoh Aktifitas Fisik yang dapat dilakukan :

CONTOH AKTIF BERGERAK DI RUMAH	
• Mengepel dan menyapu lantai, mencuci pakaian, mengepel dll.<br>• Hindari menggunakan remote control TV<br>• Berkebun / membersihkan halaman<br>• Menggendong anak, mendorong kereta bayi, berjalan di taman	• Mencuci pakaian, mencuci mobil, mengepel lantai, membawa belanjaan, dll<br>• Bermain aktif bersama anak di dalam dan luar rumah seperti petak umpet, kuda kudaan, lompat tali, bersepeda, bulu tangkis, dll

CONTOH AKTIFI BERGERAK DI TEMPAT KERJA :	
• Menggunakan tangga daripada lift untuk mencapai lantai 2-4.<br>• Mengikuti kegiatan senam bersama dikantor seperti senam jantung sehat, senam diabetes, senam osteoporosis	• Melakukan senam peregangan di kantor di sela waktu bekerja.<br>• Mengisi kegiatan rapat dengan selingan senam seperti senam cerdik, senam peregangan, dll.

Contoh aktif bergerak di tempat lain :	
• Tetap berusaha berjalan di eskalator.<br>• Manfaatkan taman kota untuk beraktifitas fisik.	• Perbanyak bermain di ruang terbuka seperti jalan, jogging.

DIET BAGI PENYANDANG OBESITAS

Selalu mengonsumsi makanan dengan pola gizi seimbang menggunakan prinsip piring makan model T untuk makanan utama dan mengutamakan konsumsi buah-buah untuk makanan selingan.

Gambar Konsep Piring Model T

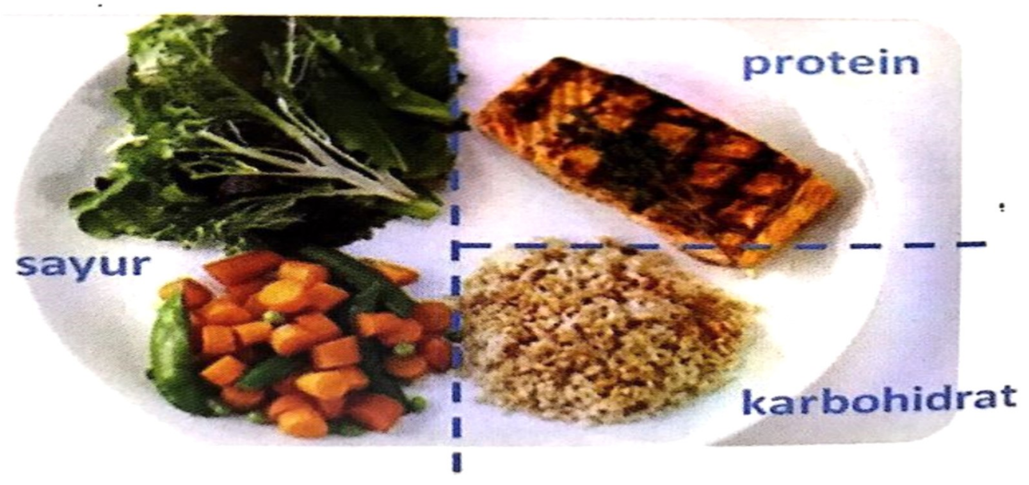

- Piring makan model T, yaitu jumlah sayur 2 kali lipat dari bahan makanan sumber karbohidrat (nasi, mie, roti, pasta dan lain-lain).
- Sayur dianjurkan 5-6 porsi.
- Jumlah buah minimal 3 porsi sama dengan jumlah karbohidrat atau protein.

6 Kegemukan (Obesitas)

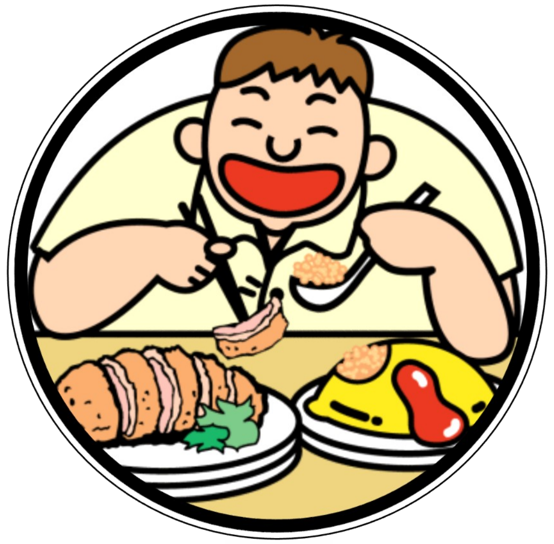

Obesitas merupakan penumpukan lemak yang ber-lebihan akibat ketidakseim-bangan asupan energi dengan energi yang digunakan dalam waktu lama.

- Seseorang dikatakan obesitas jika telah memiliki Indeks Massa Tubuh (IMT) 25 kg/m2.
- Cara perhitungan IMT:

$$IMT = \frac{\text{Berat Badan (kg)}}{\text{Tinggi Badan (m) x Tinggi Badan (m)}}$$

Penyakit yang timbul akibat obesitas:

⇒ Penyakit jantung dan stroke
⇒ Kencing manis/Diabetes
⇒ Radang tulang sendi
⇒ Kanker rahim/ payudara/ prostat/ usus besar
⇒ Nyeri pinggang
⇒ Batu empedu

Untuk mengatasi kegemukan/ obesitas dapat dilakukan upaya diantaranya :

- Diet seimbang
- Aktifitas fisik
- Pemanfaatan TOGA dan akupresur

5 DIET YANG TIDAK TEPAT

Diet Seimbang

⇒ Adalah sajian makanan yang mengandung semua nutrient utama yang diperlukan oleh tubuh pada kadar yang sesuai dengan fungsi tubuh.

⇒ Nutrien utama yang diperlukan oleh tubuh adalah Karbohidrat, protein, vitamin dan garam mineral

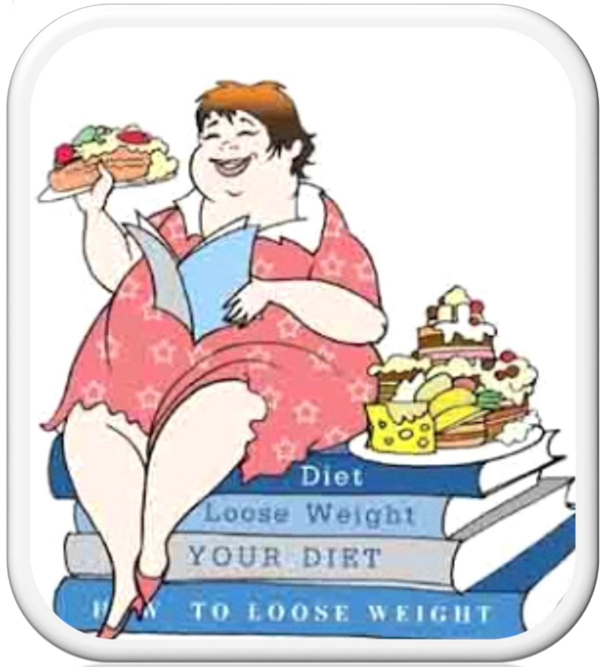

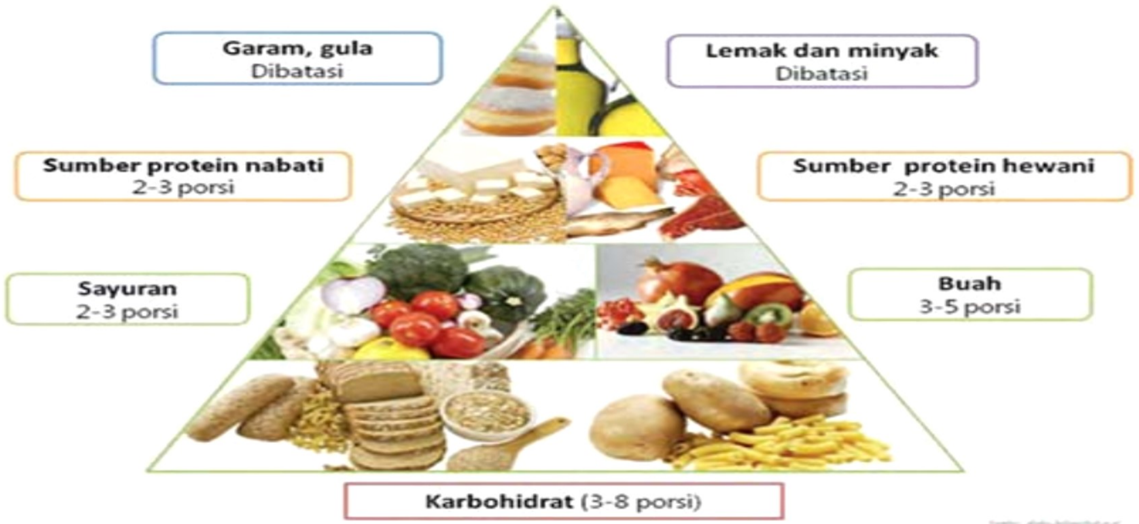

SEHAT DENGAN POLA MAKAN GIZI SEIMBANG
