# Supplementary material for: Exploring the self-efficacy and self-care-based stroke care model for risk factor modification in mild-to-moderate stroke patients
Source: Front Neurol. 2023 May 11;14:1177083. doi: 10.3389/fneur.2023.1177083 (PMC10213644; doi:10.3389/fneur.2023.1177083)
Supplement: Supplementary file 3 [file Image_2.PDF]

MODUL EDUKASI PENCEGAHAN STROKE

Untuk Perawat

Penulis :
UKE PEMILA

Kontributor :
Prof. Dr. Ratna Sitorus, SKp, M.App.SC
Agung Waluyo, SKp, M.Sc, Ph.D
Dr. Sutanto Priyo Hastono, SKM, M.Kes

Fakultas Ilmu Keperawatan
Universitas Indonesia
Tahun 2019

Kata Pengantar

Stroke merupakan masalah kesehatan yang sangat berperan sebagai penyebab kedua kematian dan penyebab ketiga kecacatan di seluruh dunia. Tercatat setiap tahunnya sekitar 5,7 juta kematian akibat stroke dan 15 juta orang terserang stroke mengalami gangguan fisik dan disabilitas. Upaya pencegahan terjadinya stroke pertama harus ditingkatkan untuk mengurangi angka kematian secara signifikan karena dari semua kejadian stroke ditemukan sebesar 77% merupakan serangan stroke pertama (Goldstein, et al dalam Price (2015), perlu dukungan multisektor untuk meningkatkan wawasan dan kesadaran masyarakat bahwa stroke harus dicegah dan faktor risikonya harus dihindari.

Modul Pencegahan Stroke ini bertujuan untuk dijadikan sebagai pedoman oleh tenaga kesehatan khususnya perawat dalam meningkatkan pengetahuan masyarakat tentang stroke dan faktor risikonya sehingga dapat meningkatkan kesadaran masyarakat akan ancaman stroke dan dapat merubah perilakunya untuk menurunkan faktor risiko stroke yang dimilikinya.

Semoga modul ini bermanfaat untuk menurunkan angka kejadian stroke di Indonesia.

Depok,   Desember 2019

Penulis

Daftar Isi

KATA PENGANTAR 1
DAFTAR ISI 2
BAB I Pengetahuan tentang stroke 3
BAB II Faktor risiko stroke 8
1. Hipertensi 9
2. Kencing Manis/Diabetes Melitus (DM) 10
3. Penyakit Jantung 19
4. Obeisitas 23
BAB III Perilaku yang harus dihindari 25
1. Merokok 26
2. Kurang Aktivitas Fisik 33
3. Kurang Konsumsi Sayur dan Buah 35
4. Konsumsi Alkohol 40
BAB IV Dukungan Sosial 41
1. Primer 42
2. Sekunder 42
3. Tertier 44
DAFTAR PUSTAKA 45

BAB I

STROKE

STROKE

Stroke adalah kejadian sakit tiba-tiba yang ditandai dengan adanya lumpuh pada sebagian sisi tubuh, bicara pelo dan dapat disertai turunnya kesadaran yang di sebabkan oleh gangguan peredaran darah ke otak akibat sumbatan atau pecahnya pembuluh darah otak. Waktu penanganan terbaik adalah 3 - 6 jam setelah timbul gejala. Dan segera dirujuk ke rumah sakit.

Gambar Stroke

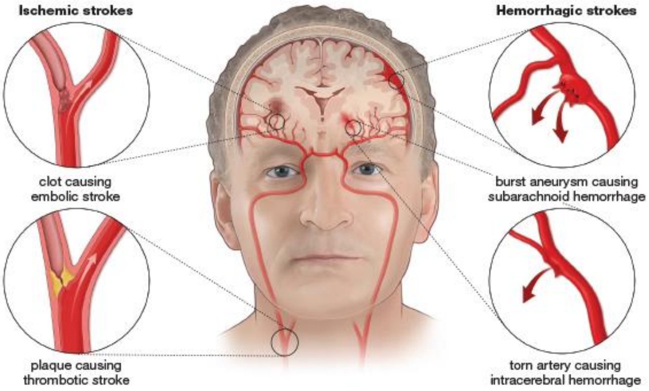

APA PENYEBAB STROKE…???

- Bekuan darah didalam pembuluh darah atau leher
- Bekuan darah atau material lain yang dibawa ke otak dari bagian tubuh yang lain
- Penurunan aliran darah ke area otak
- Pecahnya pembuluh darah otak dengan perdarahan ke dalam jaringan otak atau ruang sekitar otak

Siapa yang berisiko terkena stroke ???

a. **Faktor Risiko yang tidak bisa dirubah**
   - Usia
   - Jenis kelamin
   - Riwayat keluarga terkena stroke
   - Ras, orang kulit berwarna lebih berisiko

b. **Faktor Risiko yang dapat diubah**
   - Darah tinggi (hipertensi) sebagai faktor risiko utama
   - Penyakit Jantung
   - Kolesterol tinggi
   - Kencing Manis (Diabetes)
   - Kegemukan (Obesitas)
   - Merokok
   - Konsumsi Alkohol
   - Penyalahgunaan obat

BAHAYA STROKE

- ✓ Kematian
- ✓ Kelumpuhan
- ✓ Koma

AKIBAT STROKE

1. Gangguan gerak
2. Gangguan sensorik (pendengaran berkurang)
3. Gangguan pemahaman dan penggunaan bahasa
4. Gangguan berfikir dan memory

Stroke dengan "SEGERA KE RS" yaitu :

- Senyum yang tidak simetris
- Gerak anggota tubuh yang melemah atau tidak dapat digerakkan secara tiba-tiba
- Suara yang pelo, parau atau menghilang
- Keseimbangan dan Kesadaran Terganggu
- Rabun atau Gangguan Penglihatan tiba-tiba
- Sakit Kepala

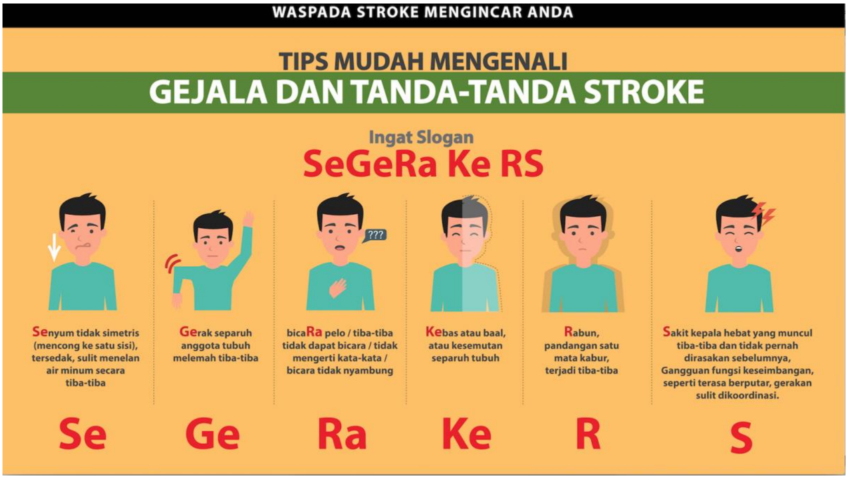

PENCEGAHAN STROKE

BAGAIMANA MENCEGAH TERJADINYA STROKE

Stroke dapat dicegah dengan mengendalikan faktor risiko yaitu :

- ✓ Kontrol Tekanan Darah, Gula darah dan kolesterol dgn : Perilaku hidup sehat seperti : konsumsi garam tidak lebih dari 1 sendok the per orang per hari, konsumsi gula tidak lebih dari 4 sendok makan per orang per hari, konsumsi lemak (minyak) tidak lebih dari 5 sendok makan perorang perhari.
- ✓ Aktifitas fisik minimal 30 menit per hari sebanyak 3-5 kali per minggu
- ✓ Tidak merokok
- ✓ Tidak mengonsumsi alkohol
- ✓ Kendalikan stres.
- ✓ Menjaga kondisi tubuh seperti : berat badan ideal, gula darah normal, kolesterol dan tekanan darah normal.

PENGENDALIAN FAKTOR RISIKO STROKE DENGAN MENERAPKAN:

PERILAKU CERDIK

C : Cek kesehatan secara berkala

E : Enyahkan asap rokok

R : Rajin aktivitas fisik

D : Diet sehat seimbang

I : Istirahat yang cukup

K : Kelola Stres

BAB II

FAKTOR RISIKO STROKE

HIPERTENSI

Hipertensi atau tekanan darah tinggi adalah suatu keadaan dimana tekanan darah sistolik ≥ 140 mmHg dan atau tekanan diastolik ≥ 90 mmmHg.

Tanda dan Gejala dan Tanda :

- Sakit kepala
- Kelelahan
- Mual dan muntah
- Sesak napas
- Napas pendek (terengah-engah)
- Gelisah
- Pandangan menjadi kabur
- Mata berkunang-kunang
- Mudah marah
- Telinga berdengung I Sulit tidur
- Rasa berat di tengkuk

DIABETES MELLITUS/ KENCING MANIS

Suatu penyakit menahun yang ditandai oleh kadar glukosa darah yang melebihi nilai normal secara menahun. Sebutan glukosa darah sering dikenal oleh masyarakat dengan gula darah.

Apa saja Tipe Penyakit DM?

a. DM tipe 1 : DM yang disebabkan tidak adanya produksi insulin sama sekali.
b. DM tipe 2 : DM yang disebabkan tidak cukup dan tidak efektifnya kerja insulin.
c. DM Gestasional : DM yang terjadi saat kehamilan.
d. DM tipe lainnya : DM tipe lain yang disebabkan oleh pemakaian obat, penyakit lain-lain, dan sebagainya.

Apa saja gejala Penyakit DM?

1. Gejala Utama (klasik)
   - Sering Kencing
   - Cepat Lapar
   - Sering haus

2. **Gejala Tambahan:**
   - Berat badan menurun cepat tanpa penyebab yang jelas.
   - Kesemutan.
   - Gatal didaerah kemaluan wanita.
   - Keputihan pada wanita.
   - Luka sulit sembuh.

- Bisul yang hilang timbul.
- Penglihatan kabur.
- Cepat lelah.
- Mudah mengantuk.
- Impotensi pada pria.

Apa saja Faktor Risiko Penyakit DM?

1. **Faktor Risiko Yang Tidak Bisa di Ubah**
   - Usia ≥40 tahun.
   - Mempunyai Riwayat keluarga menderita DM.
   - Kehamilan dengan gula darah tinggi.
   - Ibu dengan riwayat melahirkan bayi dengan (Berat Badan Lahir) > 4 kg.
   - Bayi yang memiliki Berat Badan Lahir (BBL) < 2,5 kg.

2. **Faktor Risiko Yang Bisa di Ubah**
   - Kegemukan (Berat badan lebih /IMT > 23 kg/m2) dan Lingkar Perut (Pria > 90 cm dan Perempuan >80cm).
   - Kurang aktivitas fisik.
   - Hipertensi/Tekanan darah Tinggi (> 140/90 mmHg).
   - Dislipidemia (Kolesterol HDL laki-laki ≤ 35 mg/dL dan perempuan ≤ 45, trigliserida ≥ 250 mg/dL).
   - Riwayat penyakit jantung.
   - Diet tidak seimbang (tinggi gula, garam, lemak dan rendah serat).
   - Merokok/terpapar asap rokok

Bagaimana mengetahui Penyakit DM secara dini ?

1. Mempunyai gejala utama dan atau beberapa gejala tambahan.
2. Mempunyai Faktor Risiko penyakit DM.
3. Pemeriksaan Kadar glukosa Darah menunjukkan hasil sebagai berikut:

Batas Normal Kadar Gula Darah Sewaktu adalah > 200 mg/dL Batas Normal Kadar Gula Darah Puasa adalah > 126 mg/dL

Apa yang harus dilakukan bila mengalami gejala penyakit DM?

Periksa kadar glukosa darah ke:

- Pos Pembinaan Terpadu (Posbindu PTM).
- Fasilitas Kesehatan Tingkat Pertama/FKTP (Puskesmas, Klinik Pratama).
- Fasilitas kesehatan lainnya seperti Rumah Sakit.
- Laboratorium Kesehatan.

Apa yang harus dilakukan bila telah didiagnosis penyakit DM?

1. Mengikuti Edukasi (penyuluhan dan konseling) tentang DM di :
   - Pos Pembinaan Terpadu (Posbindu PTM).
   - Fasilitas Kesehatan Tingkat Pertama/FKTP (Puskesmas, Klinik Pratama).
   - Fasilitas kesehatan lainnya seperti Rumah Sakit.
2. Mengatur pola makan sesuai dengan diet untuk penyakit DM.
3. Melakukan latihan fisik secara teratur dan tepat dengan prinsip BBTT (Baik, Benar, Terukur dan Teratur).
4. Mengonsumsi obat secara teratur sesuai petunjuk

5. Monitoring kadar glukosa darah sesuai petunjuk. Obat hanya bermanfaat bila disertai dengan pola makan yang seimbang dan latihan fisik secara teratur dan tepat

KOMPLIKASI KENCING MANIS

1. Komplikasi akut (dalam waktu cepat) dan dapat berakhir dengan kematian.
   a. Hipoglikemia (kadar glukosa darah terlalu rendah < 70 mg/dl).
      - Terjadi bila diabetesi minum obat tablet atau menggunakan obat suntik (insulin) disertai mengonsumsi makanan terlalu sedikit dan atau latihan fisik terlalu berat sehingga kadar glukosa darahnya turun terlalu rendah.
      - Mengonsumsi obat anti diabetes tidak sesuai petunjuk dokter.
      - Respon diabetesi terhadap obat anti diabetes berlebihan.
      - Gejala Hipoglikemia: Badan terasa lemas, lapar, pusing, gemetar, penglihatan kabur, keringat berlebih, kejang-kejang, kebingungan dan detak jantung yang cepat, bisa menyebabkan pingsan.

Gula Darah Rendah atau Hipoglikemi:

Adalah gangguan kesehatan yang terjadi ketika kadar gula di dalam darah berada di bawah kadar normal dan merupakan komplikasi yang paling umum terjadi pada individu dengan diabetes.

Dapat terjadi karena berbagai alasan termasuk aktifitas fisik berlebihan, penggunaan dosis insulin/obat antidiabetes atau tidak cukup makan/terlambat makan.

Tips Untuk Mengatasi Gula Darah Rendah :

1. Mengonsumsi larutan air gula atau makanan tinggi gula seperti permen.
   Larutan air gula dapat dibuat dengan cara melarutkan 2 sdm gula pasir ke dalam satu gelas air putih. Jika setelah 15 menit keluhan hipoglikemia masih tetap ada, minum kembali larutan air gula atau makanan tinggi gula.
2. Jika hasil pemeriksaan kadar glukosa darah sudah mencapai normal, maka segera mengonsumsi makanan utama atau selingan.
3. Segera berkonsultasi dengan dokter.

b. Hiperglikemia (Kadar glukosa darah sangat tinggi > 300 mg/dl).
   - Dapat menyebabkan gangguan penurunan kesadaran, mengalami infeksi yang berulang dan Penurunan Berat Badan.
   - Gejala : Sering merasa kehausan, mulut terasa kering, buang air kecil meningkat, kulit terasa kering, penglihatan menjadi buram/kabur, pusing, nafas terengah-engah dan bau nafas tak sedap.

2. Komplikasi kronis (dalam waktu lama)
   a. Gangguan ginjal
   b. Gangguan mata/penglihatan
   c. Gangguan saraf yang menyebabkan luka dan amputasi pada kaki
   d. Penyakit jantung dan pembuluh darah

Apa yang harus dilakukan agar terhindar dari komplikasi DM?

1. Atur pola makan dengan energi seimbang, disebut dengan Diet DM.
   a) Diet DM dilakukan dengan pola makan sesuai dengan aturan 3J (Jumlah, Jenis dan Jadwal Makan):
      1) Jumlah :
         a) Jumlah makanan yang dikonsumsi disesuaikan dengan BB memadai yaitu BB yang dirasa nyaman untuk seorang diabetesi.

         b) Jumlah makanan yang dikonsumsi disesuaikan dengan hasil konseling gizi.

      2) Jenis:
         a) Jenis makanan utama yang dikonsumsi dapat disesuaikan dengan konsep piring makan model T, yang terdiri dari kelompok sayuran (ketimun, labu siam, tomat, wortel, bayam, dll), karbohidrat (nasi, kentang, jagung, ubi, singkong, dll), dan protein (ikan, telur, tempe, tahu, kacang hijau, kacang merah, dll). Pengolahan sayur, karbohidrat, protein tidak meng-gunakan gula, garam dan lemak yang berlebih.
         b) Jenis makanan selingan (diantara dua waktu makan) diutamakan dari kelompok buah-buahan yang kan-dungan gulanya relatif aman yaitu pepaya, salak, melon, jeruk, bengkoang, apel, dll. Hindari buah-buahan musiman dan yang diawetkan.

3) Jadwal makan terdiri dari 3x makan utama dan 2-3x makanan selingan mengikuti prinsip porsi kecil.

2. Mengonsumsi obat secara teratur
3. Mengikuti edukasi (penyuluhan dan konseling gizi) secara berkelanjutan .
4. Mengecek kadar glukosa darah secara berkala bertujuan agar diabetesi mampu mandiri dalam mengontrol kadar glukosa darah.

TIPS HIDUP SEHAT BAGI PENYANDANG DIABETES

1.Batasi makanan berikut ini, dan sangat baik jika dapat dihindari :

a. Mengandung banyak gula sederhana

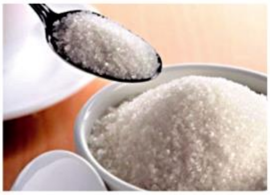
Gula Pasir

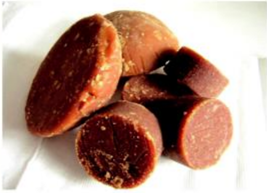
Gula Jawa/Gula Aren/ Gula Merah

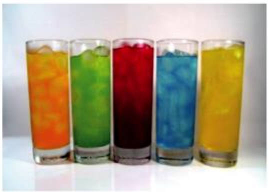
Sirup/Minuman Ringan/ Minuman Kemasan

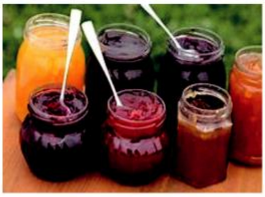
Selai diawetkan dengan gula

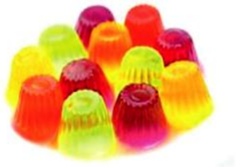
Jelly/Agar/ Puding Mas

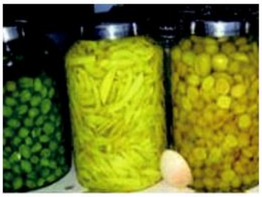
Manisan Buah/ Buah Yang

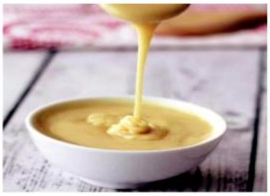
Susu Kental Manis/ Krimer Kental Manis

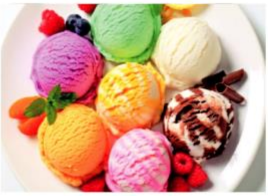
Es Krim

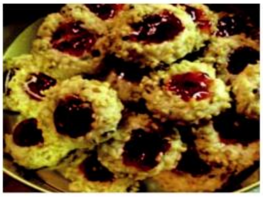
Kue-kue Manis

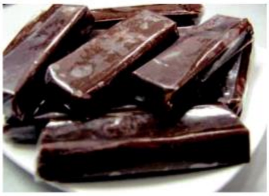
Dodol/Lempok

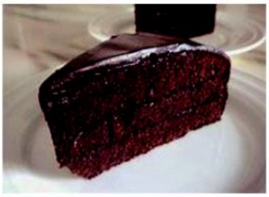
Cake/Bolu

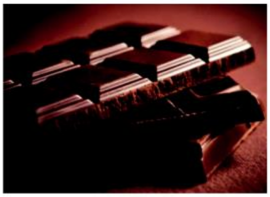
Cokelat

b. Mengandung banyak lemak

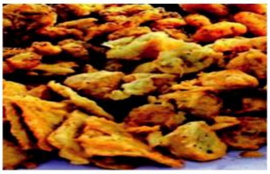

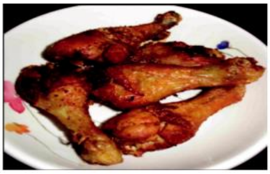

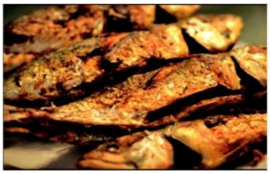

Semua makanan yang diolah dengan cara digoreng

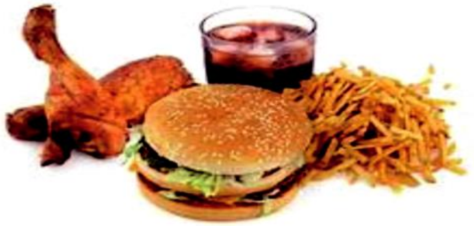

Fast Food/Makanan cepat saji

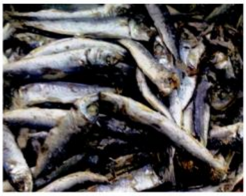

Ikan Asin

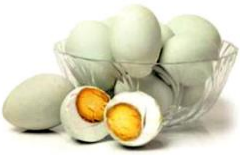

Telur Asin

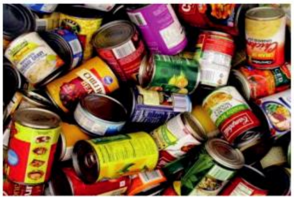

Makanan Kaleng/
Makanan yang diawetkan

2. Jika ingin mengganti gula pasir, gula aren/jawa dan gula batu dengan gula alternatif maka gunakanlah dalam jumlah terbatas. Gula alternatif yang dimaksud antara lain fruktosa, gula alkohol berupa sorbitol, manitol dan silitol, aspartame dan sakarin. Untuk mengetahuinya dapat dengan membaca label pada kemasan.

.

PENYAKIT JANTUNG

FIBRILASI ATRIUM

Adalah kondisi gangguan pada hantaran listrik jantung dan irama denyut jantung, sehingga denyut jantung tidak beraturan dan cepat. Kondisi ini akan meningkatkan risiko terjadinya penggumpalan darah, stroke dan gagal jantung.

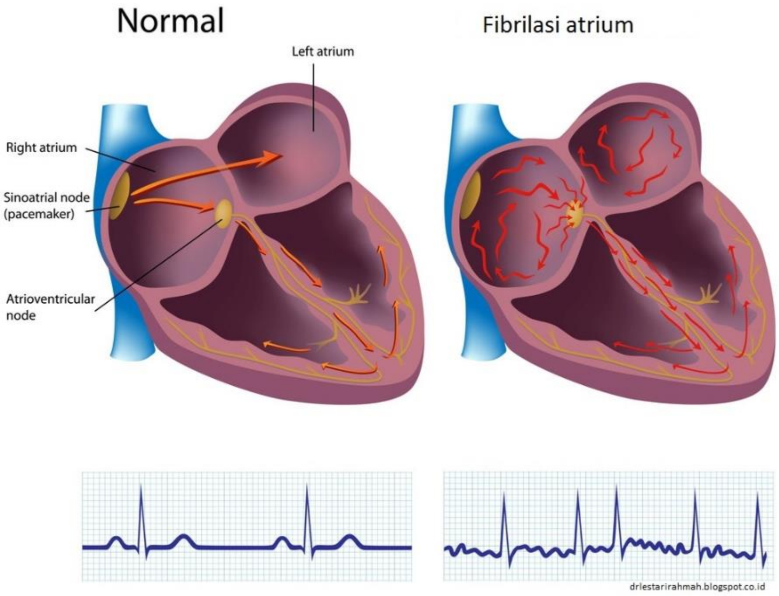

Gejala Fibrilasi Atrium :

Gejala utamanya adalah jantung berdebar atau detak jantung terasa lebih cepat, serta tidak beraturan, gejala lain seperti :

1. Kelelahan, terutama saat berolahraga
2. Pusing
3. Nafas Pendek
4. Lemah
5. Nyeri dada

Penyebab Fibrilasi Atrium :

a. Infeksi virus
b. Kelainan jantung bawaan
c. Metabolisme tidak seimbang
d. Penyakit paru paru, hipertensi dan jantung koroner
e. Paparan obat, alkohol atau tembakau
f. Gangguan pernafasan saat tidur
g. Pernah menjalani operasi jantung

h. Stress akibat penyakit atau operasi

Cara meraba nadi sendiri :

a. Genggam pergelangan tangan
b. Rabalah dengan jari telunjuk, tengah dan manis tonjolan tulang di bagian bawah pangkal ibu jari
c. Geser sedikit kea rah tengah pergelangan
d. Rasakan denyutan dan hitung dalam **30 detik**
e. Jika denyutan Nadi anda tidak teratur atau jumlah denyutan **diatas 50** atau **dibawah 30.**

WASPADA GANGGUAN IRAMA JANTUNG

Dalam keadaan normal, denyut jantung iramanya beraturan sehingga dapat mengalirkan darah antara ruang jantung yang di alirkan ke paru paru dan ke seluruh tubuh.

PENCEGAHAN FIBRILASI ATRIUM

Menurunkan risiko terjadinya fibrilasi atrium dapat dilakukan dengan menerapkan pola hidup sehat, yaitu :

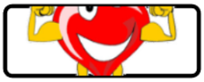	Mengkonsumsi makanan yang sehat untuk jantung, serta membatai asupan garam, lemak dan kolesterol
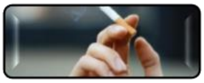	Menghentikan kebiasaan merokok
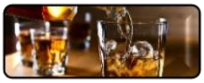	Membatasi konsumsi alkohol dan kafein
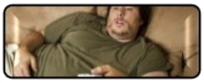	Menjaga berat badan yang normal
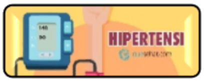	Mengendalikan tekanan darah dan kolesterol dalam darah

OBESITAS (KEGEMUKAN)

Obesitas merupakan penumpukan lemak yang berlebihan akibat ketidakseimbangan asupan energi dengan energi yang digunakan dalam waktu lama.

- Seseorang dikatakan obesitas jika telah memiliki Indeks Massa Tubuh (IMT) 25 kg/m2.
- Cara perhitungan IMT:

$$\text{IMT} = \frac{\text{Berat Badan (kg)}}{\text{Tinggi Badan (m)} \times \text{Tinggi Badan (m)}}$$

Penyakit yang timbul akibat obesitas:

- Penyakit jantung dan stroke
- Kencing manis/Diabetes
- Radang tulang sendi
- Kanker rahim/payudara/prostat/usus besar
- Nyeri pinggang
- Batu empedu
- Asma.

Untuk mengatasi kegemukan/obesitas dapat dilakukan beberapa upaya diataranya :

1. Diet seimbang
2. Aktifitas fisik
3. Pemanfaatan TOGA dan akupresur

Tips Diet Bagi Penyandang Obesitas

Selalu mengonsumsi makanan dengan pola gizi seimbang meng-gunakan prinsip piring makan model T untuk makanan utama dan mengutamakan konsumsi buah-buah untuk makanan selingan.

Gambar Konsep Piring Model T

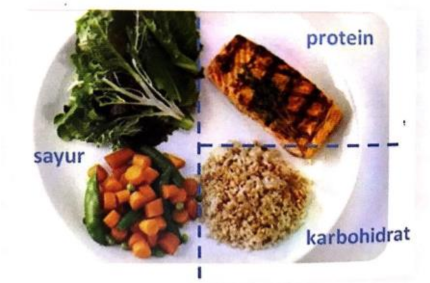

- piring makan model T, yaitu jumlah sayur 2 kali lipat dari bahan makanan sumber karbohidrat (nasi, mie, roti, pasta dan lain-lain).
- Sayur dianjurkan 5-6 porsi.
- Jumlah buah minimal 3 porsi sama dengan jumlah karbohidrat atau protein.
- Jumlah protein.

BAB III

Perilaku untuk Menghindarinya

1. **Merokok.**

   Apa itu rokok ?

   Rokok adalah hasil olahan daun tembakau dengan atau tanpa menggunakan bahan tambahan

   Jenis - jenis rokok

   1. **Rokok filter / kretek**

      Tembakau dan bahan tambahan lain yang digulung atau dilinting dengan kertas baik dengan tangan maupun menggunakaan mesin.
   2. **Cerutu**

      Tembakau murni dalam bentuk lembaran yang menyerupai rokok.
   3. **Shisha (Rokok Arab)**

      Tembakau yang dicampur dengan aroma atau perasa buah - buahan dan rempah - rempah yang dihisap dengan alat khusus.
   4. **Pipa / cangklong**

      Tembakau yang dimasukkan ke dalam pipa.
   5. **Rokok Elektronik**
      a. Istilah di pasaran : rokok elektrik, vapour, vape, electro smoke, e-cigarette, smart cigarette, personal vaporizer (pv).
      b. Suatu alat yang berfungsi seperti rokok .
      c. Tidak menggunakan atau membakar daun tembakau.
      d. Mengubah cairan menjadi uap yang dihisap ke paru.
      e. Mengandung nikotin, zat kimia lain, perasa (flavour).
      f. Bersifat toksik (beracun).

Kandungan dalam sebatang rokok

Rokok dan produk tembakau lainnya yang dikonsumsi manusia umumnya merupakan daun tanaman tembakau (Nicotiana tabacum, Nicotiana rustica). Di dalam daun tembakau olahan, terdapat 2.550 bahan kimia. Beberapa bahan kimia tersebut cepat menimbulkan gangguan kesehatan, kerusakan paru, dan dapat melemahkan stamina tubuh.

Bila daun tembakau tersebut diolah menjadi sebatang rokok, maka di dalamnya terkandung lebih dari :

a. 4000 jenis senyawa kimia
b. 400 zat berbahaya
c. 43 zat penyebab kanker (karsinogenik).

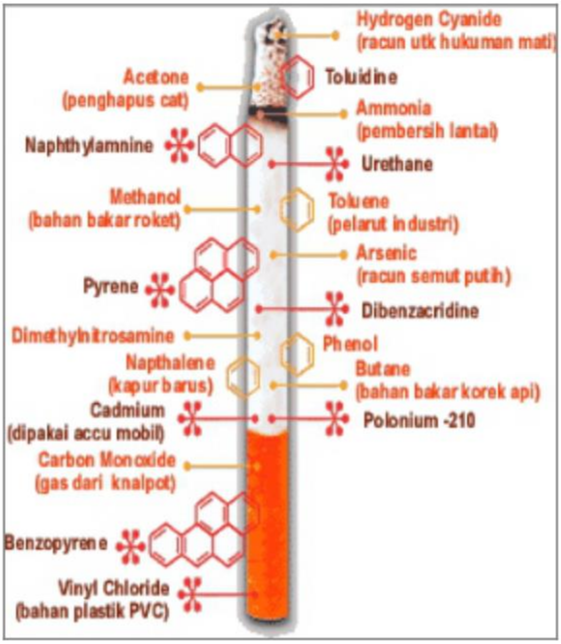

Zat berbahaya dalam sebatang rokok

Kandungan racun yang paling utama di dalam sebatang rokok, berupa :

a. **Nikotin**
   - Zat berbahaya
   - Penyebab kecanduan (adiksi)
b. **Carbon Monoksida (CO)**
   - Gas yang beracun
   - Hasil pembakaran tidak sempurna
   - Menurunkan kadar oksigen dalam darah
c. **Tar**
   - Zat berbahaya
   - Bersifat lengket
   - Menempel pada paru - paru
   - Mengandung bahan - bahan karsinogenik (penyebab kanker)

Mengapa harus berhenti merokok?

Merokok merupakan salah satu faktor risiko bersama PTM sehingga perlu dihindari. Berikut merupakan penjelasan tentang beberapa hal yang perlu diperhatikan berkaitan dengan rokok.

Bahaya merokok

Bahaya terhadap kesehatan individu dibedakan atas 1). perokok aktif yakni perokok itu sendiri dan 2). perokok pasif yakni orang lain yang tidak merokok namun menghirup asap rokok.

Merokok dapat menyebabkan:

a. Tekanan darah tinggi, penyakit jantung, stroke.
b. Asma, penyakit paru obstruktif kronik.
c. Tukak lambung, radang usus besar.

d. Kanker paru, kanker payudara, kanker lambung, kanker usus.

e. Mandul/Infertilitas, impotensi, gangguan pada kehamilan dan janin.
f. Cacat bawaan

Manfaat berhenti merokok

Manfaat yang diperoleh, berupa manfaat dari sisi kesehatan, sosial, dan ekonomi. Manfaat berhenti merokok dari sisi kesehatan dapat dilihat pada tabel di bawah ini :

MULAI BERHENTI MEROKOK	MANFAAT
20 menit	Tekanan darah, denyut jantung, dan aliran darah tepi membaik
12 jam	- Hampir semua nikotindalam tubuhsudah dimetabolisme<br>- Kadar COdi dalam darah kembalinormal
24 - 48 jam	- Nikotinmulai tereliminasi dari tubuh<br>- Fungsi pengecapdan penciumanmulai membaik<br>- Sistem kardiovaskular meningkat baik
5 hari	- Sebagianbesar metabolitnikotin dalam tubuh Sudah hilang<br><br>- Fungsi pengecapdan penciumanmenjadi lebih membaik

MULAI BERHENTI MEROKOK	MANFAAT
2 - 6 minggu	- Risiko infeksi pada luka setelahoperasi berkurang secara bermakna<br>- Fungsi silia (rambutgetar) pada saluran napas dan fungsi paru membaik<br>- Napas lebih lega dan batuk-batuk berkurang
1 tahun	Risiko penyakit jantung koroner menurun setengahnya disbanding orang yang tetap merokok
5 tahun	Risiko strokemenurun pada level yang sama seperti orang tidak pernah merokok
10 tahun	Risiko kanker paru berkurangsetengahnya
15 tahun	Semua penyebab mortalitas (kematian) dan risiko Penyakit jantung koroner menurun pada level yang sama seperti orang yang tidak pernah merokok

Ditinjau dari sisi ekonomi, berhenti merokok akan memberikan peluang lebih besar dalam mengalokasikan uang atau penghasilan untuk menyediakan makanan bergizi bagi keluarga, pemenuhan kebutuhan hidup sehari-hari (pakaian, air, listrik, telepon, dst), pendidikan, dan kesehatan.

Cara Menghindari Keinginan Merokok

1. Hindari berkumpul dengan teman - teman yang sedang merokok
2. Yakinlah, bahwa rokok bukan satu - satunya sarana pergaulan
3. Jangan malu mengatakan bahwa diri kita bukan perokok
4. Perbanyak mencari informasi tentang bahaya rokok
5. Hindari sesuatu yang terkait tentang rokok (sponsor, iklan, poster, rokok gratis).
6. Lakukan hal - hal positif lainnya, seperti olah raga, membaca, berkebun, atau hobi lain yang menyehatkan.

Tips berhenti merokok

1. **Motivasi**
   Bulatkan tekad dan tujuan Anda berhenti merokok.
2. **Metode berhenti**
   Berhenti seketika (total), menunda merokok pertama, atau mengurangi jumlah rokok per hari secara bertahap.
3. **Tahan diri**
   Menahan keinginan merokok dengan menunda merokok.
4. **Olah raga**
   Berolah raga secara teratur, minimal 3x seminggu selama 15 - 30 menit.
5. **Dukungan**
   Mintalah dukungan dari keluarga dan sahabat.
6. **Konsultasi**
   Konsultasikan dengan Dokter atau tenaga kesehatan lainnya.

Menciptakan rumah tanpa asap rokok

Peran keluarga sangat diperlukan dalam menciptakan Rumah Tanpa Asap Rokok.

Peran yang bisa dilakukan :

1. Membuat kesepakatan keluarga untuk menciptakan rumah tangga tanpa asap rokok.
2. Menegur anggota keluarga yang merokok di dalam rumah.
3. Tidak memberi dukungan kepada orang yang merokok dalam bentuk apapun, antara lain dengan tidak memberikan uang untuk membeli rokok, tidak memberikan kesempatan untuk merokok di dalam rumah, tidak menyediakan asbak.
4. Tidak menyuruh anak membelikan rokok.
5. Orang tua menjadi panutan dalam perilaku tidak merokok
6. Melarang anak merokok

2. Kurang aktivitas fisik

Upaya kesehatan olahraga untuk meningkatkan kesehatan dan kebugaran jasmani dilakukan melalui aktivitas fisik, latihan fisik dan kebugaran jasmani.

Pengertian:

Aktivitas fisik merupakan setiap gerakan tubuh yang dapat meningkatkan pengeluaran tenaga atau energi. Aktivitas fisik dapat dilakukan di berbagai situasi dan tempat.

Contoh Aktif Bergerak di Rumah

• Mengepel dan menyapu lantai, mencuci pakaian, mengepel dll.<br>• Hindari menggunakan remote control TV<br>• Berkebun / membersihkan halaman<br>• Menggendong anak, mendorong kereta bayi, berjalan di taman	• Mencuci pakaian, mencuci mobil, mengepel lantai, membawa belanjaan, dll<br>• Bermain aktif bersama anak di dalam dan luar rumah seperti petak umpet, kuda kudaan, lompat tali, bersepeda, bulu tangkis, dll

Contoh Aktif Bergerak di Tempat Kerja

• Menggunakan tangga daripada lift untuk mencapai lantai 2-4.<br>• Mengikuti kegiatan senam bersama dikantor seperti senam jantung sehat, senam diabetes, senam osteoporosis	• Melakukan senam peregangan di kantor di sela waktu bekerja.<br>• Mengisi kegiatan rapat dengan selingan senam seperti senam cerdik, senam peregangan, dll.

Contoh Aktif Bergerak di tempat lain

• Tetap berusaha berjalan di eskalator.<br>• Manfaatkan taman kota untuk beraktifitas fisik.	• Perbanyak bermain di ruang terbuka seperti jalan, jogging.

Manfaat aktivitas fisik:

- ✓ Menjaga tubuh dalam kondisi yang optimal untuk menjalankan aktivitas keseharian.
- ✓ Mengurangi keluhan yang timbul akibat terlalu banyak duduk, berdiri terlalu lama atau bekerja pada posisi yang sama untuk waktu lama.
- ✓ Mencegah kegemukan, penyakit jantung pembuluh darah, stroke, diabetes dan penyakit tidak menular lain.
- ✓ Menjaga kondisi tubuh tetap bugar dan sehat.
- ✓ Menjaga tekanan darah tetap stabil dalam batas normal.
- ✓ Meningkatkan daya tahan tubuh terhadap penyakit.
- ✓ Menguatkan tulang dan otot.
- ✓ Mengurangi stres, kecemasan, depresi.

**Agar mendapatkan manfaat kesehatan,
lakukan aktivitas fisik 150 menit per minggu, idealnya dilakukan 30 menit per hari**

3. Kurang Konsumsi Sayur dan Buah

Pentingnya konsumsi sayur dan buah untuk kesehatan.

Manfaat sayur dan buah:

- Sebagai sumber vitamin
- Sebagai sumber mineral
- Sebagai sumber serat
- Sebagai antioksidan.

Fungsi vitamin:

- ✓ Untuk penglihatan
- ✓ Meningkatkan daya tahan tubuh
- ✓ Antioksidan
- ✓ Melawan berbagai zat radikal di dalam tubuh
- ✓ Memelihara kesehatan jaringan penghubung di dalam tubuh kita
- ✓ Meningkatkan penyeraan zat besi dan kalsium yang kita asup.

Fungsi Mineral yang banyak terdapat dalam sayur dan buah berperan mengontrol tekanan darah.

Fungsi Serat:

Di dalam tubuh berperan untuk, untuk proses pencernaan :

- Memberikan rasa kenyang
- Mengeluarkan zat sisa yang tidak dibutuhkan tubuh.

- M
- Menjaga keseimbangan kadar glukosa darah, dan kadar lemak darah (kolesterol dan trigliserida).
- Mencegah kegemukan/ menurunkan berat badan.
- Menjaga kesehatan dan kecantikan kulit, rambut dan kuku.
- Mencegah diabetes melitus.
- Melancarkan buang air besar.
- Mencegah kanker usus.

Akibat tidak makan sayur dan buah :

- **Sulit buang air besar**
- **Gemuk dan obesitas**
- **Tekanan darah tidak terkontrol**
- **Kadar gula darah tidak terkontrol**
- **Pembuluh darah menyempit**
- **Mudah terkena penyakit menular**

Peran keluarga untuk konsumsi sayur dan buah-buahan setiap hari

✓ Mengenalkan dan mengajarkan anak-anak sejak dini untuk menyukai makan sayur dan buah setiap hari.

✓ Selalu menyediakan sayur dan buah di rumah.

Cara mengolah sayur dan buah tanpa mengurangi nutrisi

✓ Sebelum dikonsumsi, dicuci bersih minimal dengan air mengalir.

✓ Disarankan untuk konsumsi sayur dan buah yang masih segar.

✓ Sebaiknya sayur tidak dipanaskan berulang.

✓ Sayuran sekali masak langsung dihabiskan.

✓ Lebih sehat mengkonsumsi yang direbus, dikukus daripada digoreng.

Atur Pola Makan Sehat

- Menggunakan Isi Piringku Sekali Makan.

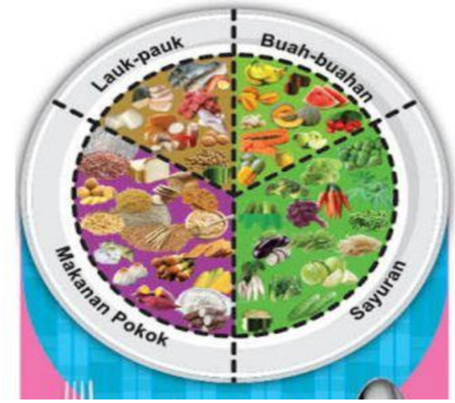

Contoh makan siang + 700 kalori

Makanan Pokok :

- 3 centong nasi.
- 3 buah kentang ukuran sedang (300gr).
- 1,5 gelas mie kering (75gr).

Lauk-pauk:

- Lauk hewani : 2 potong ayam ukuran sedang, ikan kembung (75gr), 1 butir telor ayam ukuran sedang (55gr), 2 potong daging sapi (70gr).
- Lauk nabati : tahu (100 gr), tempe ukurang sedang (50gr).

Sayuran :

- 1 mangkok sedang (150 gr).

Buah:

- Pepaya ukurang sedang (150 gr).
- 2 buah jeruk ukuran sedang (110gr).
- 1 buah pisang ambon ukuran kecil (50gr).

KEBUTUHAN SAYUR DAN BUAH :

USIA / DAUR KEHIDUPAN	JUMLAH SAYUR	JUMLAH BUAH	USIA	JUMLAH SAYUR	JUMLAH BUAH
Ibu hamil & menyusui	4 porsi	4 porsi	10-18 tahun	3 porsi	4 porsi
1-3 tahun	1.5 porsi	3 porsi	19-49 tahun	3 porsi	5 porsi
4-6 tahun	2 porsi	3 porsi	50-64 tahun	4 porsi	5 porsi
7-9 tahun	3 porsi	3 porsi	> 64 tahun	4 porsi	4 porsi

Keterangan:

1 porsi sayuran = 1 gelas tanpa air atau 100 gram, dan hanya mengandung energi 25 kkal, jadi makan sayur banyak tidak akan menggemukan.

1 porsi buah adalah:

- Apel 1 buah sedang (85 g),
- Belimbing 1 buah besar (140 g),
- Blewah 1 ptg sedang (70 g),
- Duku 9 buah (80 g),
- Jambu biji 1 buah besar (100 g),
- Jambu air 2 buah besar (110 g),
- Salak 2 buah sedang (65 g),
- Sawo 1 buah sedang (55 g),
- Semangka 1 potong besar (180 g),
- Jeruk manis 2 buah (110 g),
- Kedondong 2 buah sedang (120 g),
- Mangga ¼ buah besar (90 g),
- Melon 1 potong besar (190 g)
- Nanas ¼ buah sedang (95 g),
- Papaya 1 potong besar (110 g),
- Pisang 1 buah (50 g),
- Rambutan 8 buah (75 g),
- Sirsak ½ gelas (60 g).

TABEL KONSUMSI BUAH PER ORANG PER HARI

JENIS	UKURAN RT (1 porsi)	BERAT (gr)	UKURAN (sajian)
ALPUKAT	1/2 buah besar	50	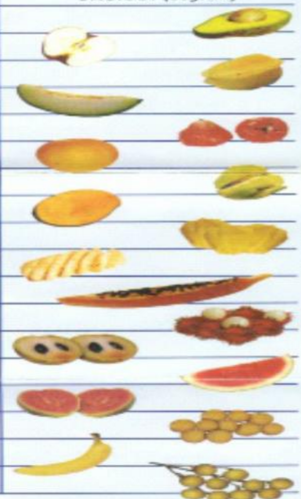
APEL	1/2 buah besar	75	
BELIMBING	1 buah besar	125	
MELON	1 potong sedang	100	
JAMBU AIR	2 buah sedang	100	
JERUK MANIS	1 buah sedang	100	
KEDONDONG	1 buah besar	100	
MANGGA	1/2 buah sedang	50	
NANGKA	3 biji	50	
NANAS	1/6 buah	75	
PEPAYA	1 potong sedang	100	
RAMBUTAN	8 buah	75	
SAWO	1 buah sedang	50	
SEMANGKA	1 potong besar	150	
JAMBU BIJI	1 buah	100	
DUKU	10 buah	75	
PISANG AMBON	1 buah	50	
LENGKENG	10 buah	75	

Jus Buah 1 porsi = 1 gelas belimbing (250 cc)

Konsumsi buah 3-5 porsi per hari

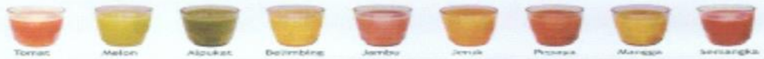

TABEL KONSUMSI SAYURAN PER GRAM PER HARI

JENIS SAYURAN	BERAT (gram)	CARA MEMASAK	UKURAN RT	SAJIAN / PORSI (URT)
BAYAM	100 gr	DITUMIS<br>atau<br>DIREBUS<br>atau<br>DIKUKUS<br>atau<br>LALAPAN	1 piring kecil (Diameter 11 cm)<br>atau<br>1 mangkuk kecil (Diameter 10 cm)<br>atau<br>1 sendok panci<br>atau<br>2 sendok sayur<br>atau<br>4 sendok bebek<br>atau<br>5 sendok makan	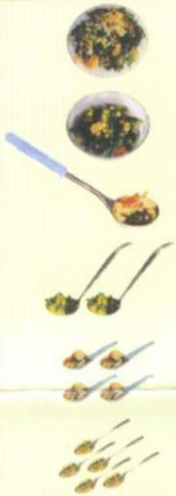
BUNCIS	100 gr			
BUNGA KOL	100 gr			
CABE HIJAU	100 gr			
DAUN SINGKONG	100 gr			
DAUN PEPAYA	100 gr			
DAUN BAWANG	100 gr			
DAUN MELINJO	100 gr			
DAUN PAKIS	100 gr			
JAGUNG MUDA	100 gr			
JAMUR SEGAR	100 gr			
KANGKUNG	100 gr			
LABU SIAM	100 gr			
LOBAK	100 gr			
OYONG	100 gr			
PARE	100 gr			
PEPAYA MUDA	100 gr			
REBUNG	100 gr			
SAWI	100 gr			
TAUGE	100 gr			
TERONG	100 gr			
TOMAT	100 gr			
WORTEL	100 gr			
KACANG PANJANG	100 gr			
KETIMUN	100 gr			

Konsumsi sayuran 2-3 porsi per hari

4. Konsumsi alkohol

Bahaya konsumsi alkohol:

- ✓ Menyebabkan gangguan penurunan fungsi seperti penilaian yang buruk.
- ✓ Reaksi pengambilan keputusan lama.
- ✓ Kewaspadaan yang rendah.
- ✓ Penurunan tajam penglihatan.

Berikut ini adalah dampak alkohol terhadap kesehatan :

- Melemahkan otot jantung dan meningkatkan risiko serangan jantung, stroke dan hipertensi.
- Peradangan pankreas.
- Kerusakan otak.
- Infeksi paru-paru.
- Kerusakan hati.
- Kerusakan ginjal.

BAB IV

Meningkatkan Dukungan Sosial

MENINGKATKAN DUKUNGAN SOSIAL

Dukungan social terbagi dlm 3 yaitu, primer, sekunder dan tertier. Dukungan primer adalah dukungan dari orang tua, pasangan dan anggota keluarga lainnya, dukungan sekunder adalah dukungan dari teman sebaya, teman kerja sedangkan dukungan tertier adalah berupa dukungan dari pimpinan, instansi atau suatu kebijakan yang memiliki peran yang bisa memberikan dampak positif terhadap kesehatan dan kesejahteraan psikologis penderita dengan penyakit kronis seperti hipertensi, diabetes, kolesterol, dll. Dukungan dari pihak lain ini menjadi kunci agar penderita penyakit kronis tidak sampai mengalami komplikasi yang bisa mengancam jiwanya.

Aspek dukungan sosial yang diberikan adalah :

a. Dukungan emosional, yaitu mencakup ungkapan empati, kepedulian, dan perhatian terhadap orang yang bersangkutan.
b. Dukungan penghargaan, yaitu terjadi lewat ungkapan hormat (penghargaan) positif bagi orang itu, dorongan maju atau persetujuan dengan gagasan atau perasaan individu, dan perbandingan positif orang itu dengan orang lain.

c. Dukungan instrumental, yaitu mencakup bantuan langsung untuk mempermudah perilaku yang secara langsung untuk mempermudah perilaku secara langsung menolong individu. Misalnya bantuan benda, pekerjaan, dan waktu.
d. Dukungan informatif, yaitu mencakup pemberian nasehat, saran-saran, atau umpan balik.

1. PRIMER

- Mendiskusikan secara terbuka sebagai bagian dari membangun dukungan keluarga.
- Meningkatkan kepatuhan minum obat
- Mengubah kebiasaan makan
- Mengajak serta untuk beraktifitas bersama
- Menyepakati rumah bebas asap rokok
- Mengajak untuk kontrol kesehatan

2. SOSIAL (*Peer Support*)

Yang termasuk dalam dukungan sosial adalah :

a. Kelompok dan Masyarakat
b. Teman sebaya/teman kerja
c. Nilai Budaya

Dukungan yang dapat didapatkan dari teman sebaya/teman kantor :

- Mencari teman yang hampir sama kondisinya, agar saling menguatkan atau saling memotivasi.
- Diskusikan dengan teman harapan terhadap kesehatan, agar teman dapat saling mengingatkan

3. KEBIJAKAN

- Mengikuti kebijakan2 di tempat kerja yang mendukung kesehatan.
- Mengikuti kebijakan pemerintah dalam GERMAS
- Melakukan pengelolaan penyakit dengan CERDIK
- Mencanangkan porsi makan dengan PIRINGKU
- Menetapkan area2 dilarang merokok dan sanksi bila merokok
- Program Program Indonesia Sehat dengan Pendekatan Keluarga (PIS-PK)

DAFTAR PUSTAKA

- Kemenkes RI. 2017. Panduan Pelaksanaan Gerakan Nusantara Tekan Angka Obesitas (GENTAS). Kemenkes RI
- Kemenkes RI 2016. Pedoman Pengendalian Obesitas. Jakarta. Kemenkes RI
- Permenkes Nomor 71 Tahun 2015 tentang Penanggulangan Penyakit Tidak Menular
- Kemenkes RI, Petunjuk Teknis Upaya Berhenti Merokok pada Fasyankes Primer, Jakarta, 2016
- Kemenkes RI, Hidup Sehat Tanpa Rokok, Jakarta, 2017
- Kemenkes RI, Pedoman Penyakit Terkait Rokok, Jakarta, 2018
- http://p2ptm.kemkes.go.id/infographic-p2ptm/obesitas/isi piringku-sekali-makan.
